# RovC - a novel type of hexameric transcriptional activator promoting type VI secretion gene expression

**DOI:** 10.1371/journal.ppat.1008552

**Published:** 2020-09-23

**Authors:** Vanessa Knittel, Pooja Sadana, Stephanie Seekircher, Anne-Sophie Stolle, Britta Körner, Marcel Volk, Cy M. Jeffries, Dmitri I. Svergun, Ann Kathrin Heroven, Andrea Scrima, Petra Dersch

**Affiliations:** 1 Department of Molecular Infection Biology, Helmholtz Centre for Infection Research, Braunschweig, Germany; 2 Young Investigator Group Structural Biology of Autophagy, Department of Structure and Function of Proteins, Helmholtz Centre for Infection Research, Braunschweig, Germany; 3 Institute for Infectiology, Center for Molecular Biology of Inflammation (ZMBE), University of Münster, Germany; 4 European Molecular Biology Laboratory, Hamburg Unit, Hamburg, Germany; 5 German Center for Infection Research, Baunschweig, Germany; Tufts University, UNITED STATES

## Abstract

Type VI secretion systems (T6SSs) are complex macromolecular injection machines which are widespread in Gram-negative bacteria. They are involved in host-cell interactions and pathogenesis, required to eliminate competing bacteria, or are important for the adaptation to environmental stress conditions. Here we identified regulatory elements controlling the T6SS4 of *Yersinia pseudotuberculosis* and found a novel type of hexameric transcription factor, RovC. RovC directly interacts with the T6SS4 promoter region and activates T6SS4 transcription alone or in cooperation with the LysR-type regulator RovM. A higher complexity of regulation was achieved by the nutrient-responsive global regulator CsrA, which controls *rovC* expression on the transcriptional and post-transcriptional level. In summary, our work unveils a central mechanism in which RovC, a novel key activator, orchestrates the expression of the T6SS weapons together with a global regulator to deploy the system in response to the availability of nutrients in the species' native environment.

## Author summary

A unique feature of type VI secretion systems is that several pathogenic bacteria encode multiple clusters for evolutionary distinct T6SSs, which presumably play a different role in their lifestyle. Some are necessary during infection for full virulence towards mammalian hosts, whereas others are needed for resistance to a broad range of adverse stresses and/or interbacterial competition in multibacterial communities by delivering bacteriolytic effectors into competing bacterial cells. Here we provide new insights into the control factors and regulatory circuits involved in a T6SS of *Yersinia*, which is activated under certain conditions to promote persistence and interbacterial competition in encountered host or environmental niches. Specifically, we identified a novel type of transcriptional activator that is able to induce T6SS expression. Based on the complex regulatory network, which fine-tunes its expression, it appears that this regulatory device has evolved as a switch that allows the rapid production of the T6SS apparatus only whenever it is needed during their environmental and host-associated lifestyle.

## Introduction

The type VI secretion system (T6SS) is a complex, versatile multiprotein nanomachine, which is dedicated to the delivery of toxic effectors into prokaryotic and eukaryotic cells. Depending on the bacterial species and their ecological niche, it thus participates in inter- and intrabacterial competition as well as bacterial pathogenesis [1, 2, 3]. The T6SS is widespread in about 25% of sequenced Gram-negative bacteria and forms an injection apparatus resembling the contractile tail of T4 bacteriophages [1, 4, 5, 6, 7]. The core components of the apparatus are usually encoded by a single gene cluster on the bacterial chromosome [7, 8, 9, 10], and include: (I) a tail tube formed by Hcp hexamers with (II) a spike-like tip of PAAR and VgrG proteins, (III) the TssBC/VipAB sheath, (IV) the TssJLM membrane complex, spanning the inner and outer membrane, and (V) the baseplate comprising the TssAEFGK proteins [4, 11, 12, 13]. Upon target cell contact, the sheath contracts and the effector-decorated tip complex is propelled outwards with the tail tube to pierce the target cell membrane and inject the toxic effector proteins [1, 13]. Bacteria predominantly use their T6SSs to eradicate competitors, although some eukaryotic targets are also known; e.g. *V*. *cholerae* exerts its T6SS-mediated toxicity towards amoebae and macrophages, and *Serratia marcescens* against fungi such as *Candida albicans* [3].

Numerous T6SS-positive bacteria contain not only one but several (up to seven) distinct copies of T6SS gene clusters [7, 10]. It is assumed that this multiplicity corresponds to specific roles and functions for defined niches in the lifestyles of the bacteria [1, 14]. Multiple T6SS gene clusters were also identified in human pathogenic *Yersinia* species. The closely related *Yersinia* species, *Y*. *pestis*, the causative agent of plague and *Y*. *pseudotuberculosis*, an enteric pathogen, which causes a range of gut-associated diseases (Yersiniosis) encode 4–6 conserved clusters of which one is smaller and most likely represents a partial cluster [9, 15]. A phylogenetic analysis revealed three different types of T6SS in *Y*. *pseudotuberculosis*. Two copies (T6SS3, T6SS4) show strong homology to T6SSs that are implicated in inter-/intrabacterial competition. Another (T6SS1) resembles the T6SS of *Vibrio cholerae* shown to have cytotoxic effects against unicellular organisms/protists and macrophages, whereas the function of the fourth type (T6SS2) is still unknown [15]. The individual function of the different system types is far from being understood, and their potential role and need for the adaptation to distinct environments and host niches is still unclear. Initial studies analyzing the expression control of the systems revealed multiple different, common as well as specific regulatory cues that ensure their appropriate expression [1, 4].

**Fig 1:**
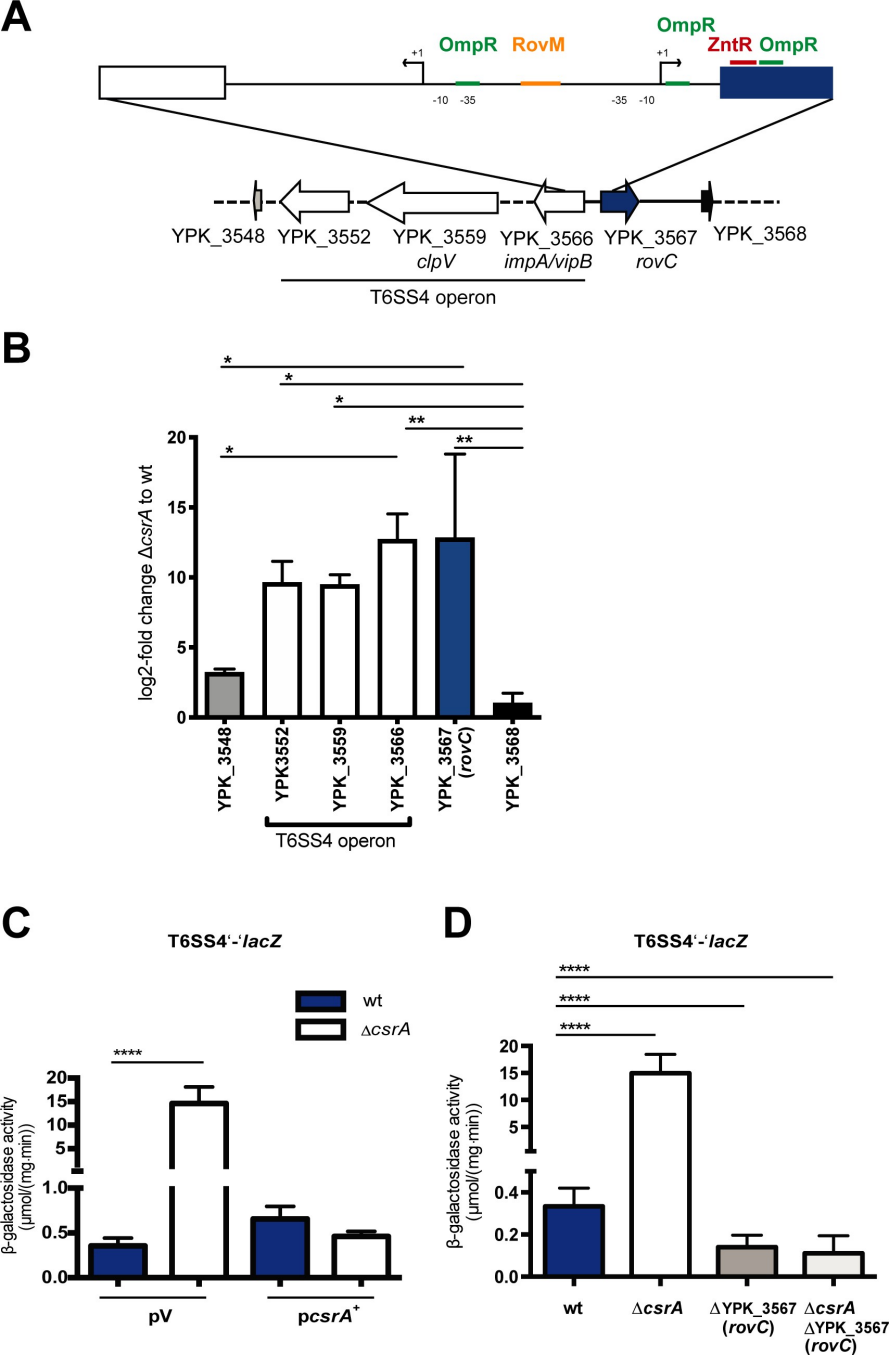
CsrA represses the expression of the T6SS4 gene cluster through regulation of *rovC*. (A) Schematic representation of the T6SS4 gene cluster of *Y*. *pseudotuberculosis*. The cluster (composed of 16 genes YPK_3550 to YPK_3566), encompassing a size of 23.6 kb is indicated with white arrows representing the tested T6SS4 genes. The *rovC* gene is found directly downstream of this cluster oriented in the opposite direction and is given by a blue arrow. The independently controlled up- and downstream genes are indicated in grey and black. (B) A qRT-PCR was performed with total RNA isolated from *Y*. *pseudotuberculosis* strain YPIII (wildtype) and the isogenic **Δ***csrA* mutant (YP53) with primer pairs specific for selected genes of the T6SS4 operon and genes upstream and downstream of the cluster. Gene expression levels were normalized to the *sopB* reference transcript according to Pfaffl 2001 [70] and log2 fold changes of **Δ***csrA* to wildtype were determined. The data represent the mean ± standard deviation of three independent experiments carried out in duplicate. Data were analyzed by one-way ANOVA, followed by Tukey’s test; *P < 0.0332, **P < 0.0021 (C) β-galactosidase activity (μmol/mg^.^min) expressed by a translational T6SS4’-‘*lacZ* reporter fusion (pSSE64) was monitored in the wildtype strain YPIII and the Δ*csrA* mutant strain YP53 (Δ*csrA*) harboring the empty vector pAKH85 (pV) and the *csrA*^+^ plasmid pAKH56 (p*csrA*^+^) grown over night for 16 h at 25°C in LB medium. The data represent the mean ± standard deviation of three independent experiments, carried out in triplicates. Data were analyzed by Student’s t-test; ****P < 0.0001; *P < 0.05. (D) To analyze the role of *rovC* in T6SS4 regulation, expression of a translational T6SS4’-‘*lacZ* reporter fusion (pSSE64) was measured in the *Y*. *pseudotuberculosis* wildtype (YPIII), the Δ*csrA* mutant (YP53), the Δ*rovC* mutant (YP148) and the Δ*csrA/*Δ*rovC* double mutant (YP318). β-galactosidase activity (μmol/mg^.^min) was measured of strains grown over night for 16 h at 25°C in LB medium. The data represent the mean ± standard deviation of three independent experiments, carried out in triplicates. Data were analyzed by Student’s t-test; ****P < 0.0001.

Because the assembly, contraction, (dis)assembly cycle of a functional T6SS organelle is energetically highly costly, it is not surprising that in most pathogenic strains, including those of *Y*. *pseudotuberculosis*, the T6SS gene clusters are silent or only weakly expressed under standard laboratory growth conditions but respond to certain environmental or in-host conditions [4, 16]. Most research, however, has been devoted to the T6SS4 cluster (**Fig 1A**), which is important for bacterial survival under acidic stress conditions. Several minor and major regulators implicated in virulence and stress regulation and different environmental parameters have been identified to trigger its expression. T6SS4 is preferentially expressed at moderate temperature during stationary phase and is subjected to quorum sensing regulation by the YpsI and YtbI synthases [17, 18]. It was further shown to be directly activated by the two-component system EnvZ/OmpR, which is responsive to osmotic, acidic and cell envelope stress [19, 20]. More recently, it was also documented that the T6SS4 cluster is part of the oxidative stress response and linked to Zn^2+^ ion acquisition, both important during pathogenesis. The oxidative stress regulator OxyR, the Zn^2+^-responsive regulator ZntR, as well as the oxidative stress-induced alternative sigma factor RpoS control T6SS4 expression [21, 22, 23]. Moreover, RovM, a LysR-type regulator, was shown to directly activate T6SS4 gene transcription by binding upstream of the T6SS4 promoter. RovM regulates important virulence genes, which contribute to host cell invasion and tissue colonization and its expression is controlled by a complex regulatory cascade which involves the carbon storage regulator (Csr) system [24, 25, 26]. This global post-transcriptional Csr system is composed of the RNA-binding protein CsrA and two antagonizing non-coding RNAs CsrB and CsrC [24, 27]. CsrA controls the expression of diverse bacterial cell functions, including virulence and stress resistance. It mostly acts by binding to GGA-motifs of its target transcripts which are part of the ribosome binding site (RBS) [28, 29, 30, 31].

In the present work, we discovered an additional, novel transcriptional activator—named RovC—of the T6SS4 gene cluster. Expression of the *rovC* gene is tightly regulated by the global nutrient-responsive regulator CsrA, and induction of its synthesis is essential for the transcriptional activation of the secretion system genes, independently of RovM. RovC is a transcriptional regulator that strongly induces expression of the T6SS4 at moderate temperatures in stationary growth phase. It folds into a novel hexameric structure with surface-exposed DNA-binding sites, which directly interact with an extended sequence in the T6SS4 promoter region.

## Results

### Identification of a CsrA-controlled activator of the T6SS4 secretion system in *Y*. *pseudotuberculosis*

In a previous study, we addressed the link between metabolism and virulence, and studied the influence of the carbon storage regulator protein CsrA on the expression of fitness- and virulence-relevant genes of *Y*. *pseudotuberculosis* [32]. Transcriptional profiling of the wildtype and a Δ*csrA* mutant revealed that all genes belonging to the T6SS4 cluster (YPK_3549 to YPK_3566, **Fig 1A**), which are transcribed from a single promoter, were upregulated in the *csrA* deficient strain. Especially, the transcript of YPK_3563, encoding Hcp was 8.71-fold more abundant in the Δ*csrA* mutant strain in comparison to wildtype [32]. A comparative expression analysis of three different genes (YPK_3552, YPK_3559, YPK_3566) of the T6SS4 cluster using qRT-PCR revealed that all genes of the cluster are strongly upregulated in the absence of CsrA compared to wildtype YPIII, whereas genes upstream (YPK_3568) and downstream (YPK_3548) of the T6SS4 operon were not influenced (**Fig 1B**). However, gene YPK_3567 encoding a hypothetical protein of 247 amino acids in the opposite direction immediately upstream of the T6SS4 gene cluster was also highly upregulated in the Δ*csrA* mutant compared to wildtype YPIII (**Fig 1B**). This implied that the T6SS4 gene cluster and the adjacent factor YPK_3567 are novel CsrA targets and we speculated that YPK_3567 could be involved in T6SS4 regulation due to its close proximity to the genetic locus of T6SS4.

To further validate the influence of CsrA and YPK_3567 on the expression of the T6SS4 gene cluster, we tested expression of a translational T6SS4’-‘*lacZ* (YPK_3566'-'*lacZ*) reporter gene fusion in the *Y*. *pseudotuberculosis* wildtype strain YPIII and its isogenic Δ*csrA* mutant. The reporter was only very weakly expressed under standard laboratory growth conditions in the wildtype. However, its expression was significantly induced in the *csrA* deficient strain, and this effect could be complemented by the introduction of a *csrA*^+^ expression plasmid (**Fig 1C**). Expression of the T6SS4’-‘*lacZ* fusion was also monitored in a ΔYPK_3567 and ΔYPK_3567 Δ*csrA* mutant. Strikingly, no induction of the T6SS4’-‘*lacZ* fusion was observed in the absence of the YPK_3567 gene (**Fig 1D**). This indicated that CsrA represses expression of the T6SS4 operon through a novel regulator encoded by YPK_3567, which acts as an activator of the T6SS4 operon. Based on the regulatory functions and properties of this novel factor, this regulator was named RovC (regulator of virulence interconnected with the Csr system).

### RovC is a novel activator of T6SS gene expression

**Fig 2:**
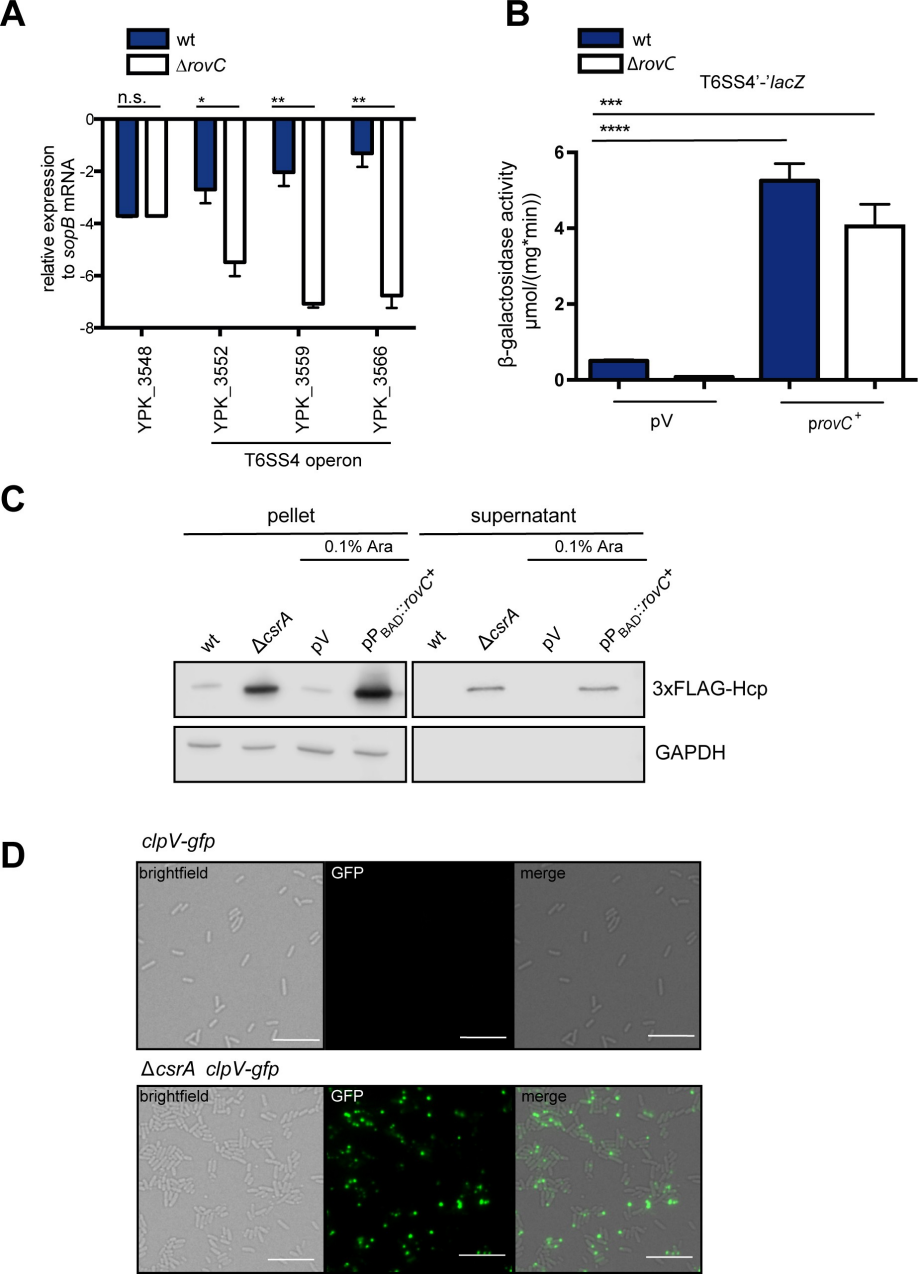
RovC activates expression of the T6SS4 operon. (A) RT-PCR analysis of selected genes of the T6SS4 cluster region was performed with total RNA isolated from at least three independent cultures of *Y*. *pseudotuberculosis* wildtype (YPIII) and the Δ*rovC* mutant strain (YP148). Expression levels were normalized to the *sopB* reference transcript according to Pfaffl 2001 [70] and are given as relative expression of the respective gene in relation to *sopB*. Data are given as means ± standard deviation and were analyzed by Student's t test. Asterisks indicate the results that differed significantly with ** P < 0.01, * P < 0.05, n.s. = not significant. (B) β-galactosidase activity (μmol/mg^.^min) of a translational T6SS4’-‘*lacZ* reporter fusion (pSSE64) was monitored in *Y*. *pseudotuberculosis* wildtype (YPIII) and the Δ*rovC* mutant (YP148) harboring the empty vector pACYC184 (pV) or the *rovC*^+^ plasmid pSSE11 (p*rovC*^+^) grown over night at 25°C. The data represent the mean ± standard deviation of three independent experiments, carried out in triplicates. Data were analyzed by Student’s t-test; ****P < 0.0001; ***P < 0.001. (C) Expression and secretion of the T6SS4 tube protein Hcp was analyzed with *Y*. *pseudotuberculosis* YPIII (wt), YP53 (Δ*csrA*), YPIII pBAD30 (pV) and YPIII pVK25 (pP_BAD_::*rovC*^+^) harboring a chromosomally-encoded gene for the N-terminal 3x FLAG-tagged Hcp protein. Strains were grown in LB at 25°C +/- 0.1% arabinose and whole cell extracts and supernatant samples were prepared, separated on 15% polyacrylamide gels and 3x-FLAG-Hcp and GAPDH were detected by western blotting using a monoclonal α FLAG and polyclonal GAPDH antibody. A representative image of three independent experiments is shown. (D) T6SS4-activity in *Y*. *pseudotuberculosis* strains YP412 (*clpV-gfp*) or YP417 (*clpV-gfp*, Δ*csrA*) grown in LB at 25°C was monitored by the identification and location of the T6SS4-associated ClpV-GFP protein using fluorescence microscopy. Cultures were grown in LB to reach an OD_600_ of 2.5 in LB. A representative image of three independent experiments is shown. The scale bar represents 10 μm.

Next, we validated whether RovC is required for the activation of T6SS4 gene expression. To do so, RNA was isolated from the *Y*. *pseudotuberculosis* wildtype strain YPIII and the Δ*rovC* mutant and a qRT-PCR analysis was performed to monitor the transcript levels of three different genes of the T6SS4 operon (YPK_3552, YPK_3559, YPK_3566), and a control transcript encoded by a gene (YPK_3548) in the close vicinity of the T6SS4 gene cluster (**Figs 1A and 2A**). The abundance of the transcripts of all tested T6SS4 components was significantly reduced in the absence of RovC, whereas the unrelated YPK_3548 transcript was not affected, suggesting that RovC is a positive regulator specific for the T6SS4 operon (**Fig 2A**). Influence of RovC on T6SS4 gene expression was further analyzed by monitoring the expression of the T6SS4'-'*lacZ* fusion in the wildtype and the Δ*rovC* mutant (**Fig 2B**). Overall expression of the reporter was completely repressed in the absence of RovC. On the contrary, introduction of a *rovC*^+^ midi-copy plasmid in which the *rovC* gene is expressed under control of its own promoter leads to a strong induction of the T6SS4'-'*lacZ* fusion in both strains (**Fig 2B**). Taken together, these results strongly suggest that RovC is a novel regulator that is essential for T6SS4 induction.

As the functionality of T6SSs can be determined by the ability to export the Hcp protein into the supernatant [33], we also assessed RovC-dependent secretion of a chromosomally-encoded FLAG_3_-tagged Hcp by T6SS4 in the wildtype, the Δ*csrA* mutant, or the *rovC* overexpression strain. As shown in **Fig 2C**, FLAG_3_-tagged Hcp was only detectable in the supernatant when RovC production was induced or the *csrA* gene was deleted. In agreement with previous results, a considerably lower amount of FLAG_3_-tagged Hcp was found in pelleted wildtype cells in the absence of the *rovC*^+^ plasmid and with wildtype levels of CsrA, and no Hcp protein was detectable in the supernatant. Absence of the cytosolic protein GAPDH in the supernatants further demonstrated that the bacterial cells remained intact. This indicates that synthesis of the T6SS4 components is low under standard growth conditions but can be triggered by induction of *rovC* expression from a plasmid or by the deletion of *csrA*. In addition, we confirmed functionality of the T6SS4 by microscopy (**Fig 2D**).

A functional and dynamic T6SS can be visualized by fusion of a fluorescent protein to one of the T6SS components. For instance, a contracted sheath, which is disassembled by the protease ClpV, can be detected by a ClpV-GFP reporter fusion as foci at the locus where a T6SS apparatus is disassembled [34]. To detect functional T6SS4, we constructed reporter strains of the wildtype (YP412) and the RovC-overexpressing Δ*csrA* mutant (YP417) with a chromosomally-encoded *clpV*_T6SS4_*-gfp* fusion and perfomed fluorescence microscopy. We could locate the ClpV_T6SS4_-GFP protein in focal points, as expected for a functional T6SS4, in the Hcp-secreting *rovC*-overexpressing and Δ*csrA* mutant strain, but not in the wildtype (**Fig 2D**). Taken together, this showed that *rovC* gene activation results in the expression of the T6SS4 locus and its functional, dynamic assembly (**Figs 1 and 2**).

### RovC interacts with the T6SS4 promoter

In order to obtain additional information regarding the potential function of RovC, we performed a BLASTp analysis of the 247 amino acid sequence of RovC of *Y*. *pseudotuberculosis* (Uniprot ID—A0A0H3B5N9) against the non-redundant protein databases. While no significant homology to proteins or protein domains with known function was detected using BLASTp, the tertiary/secondary structure prediction programs Phyre2 [35] and JPred [36] predicted a putative DNA-binding domain in the C-terminal region of RovC (185–247 aa) that folds into a helix-turn-helix motif.

**Fig 3:**
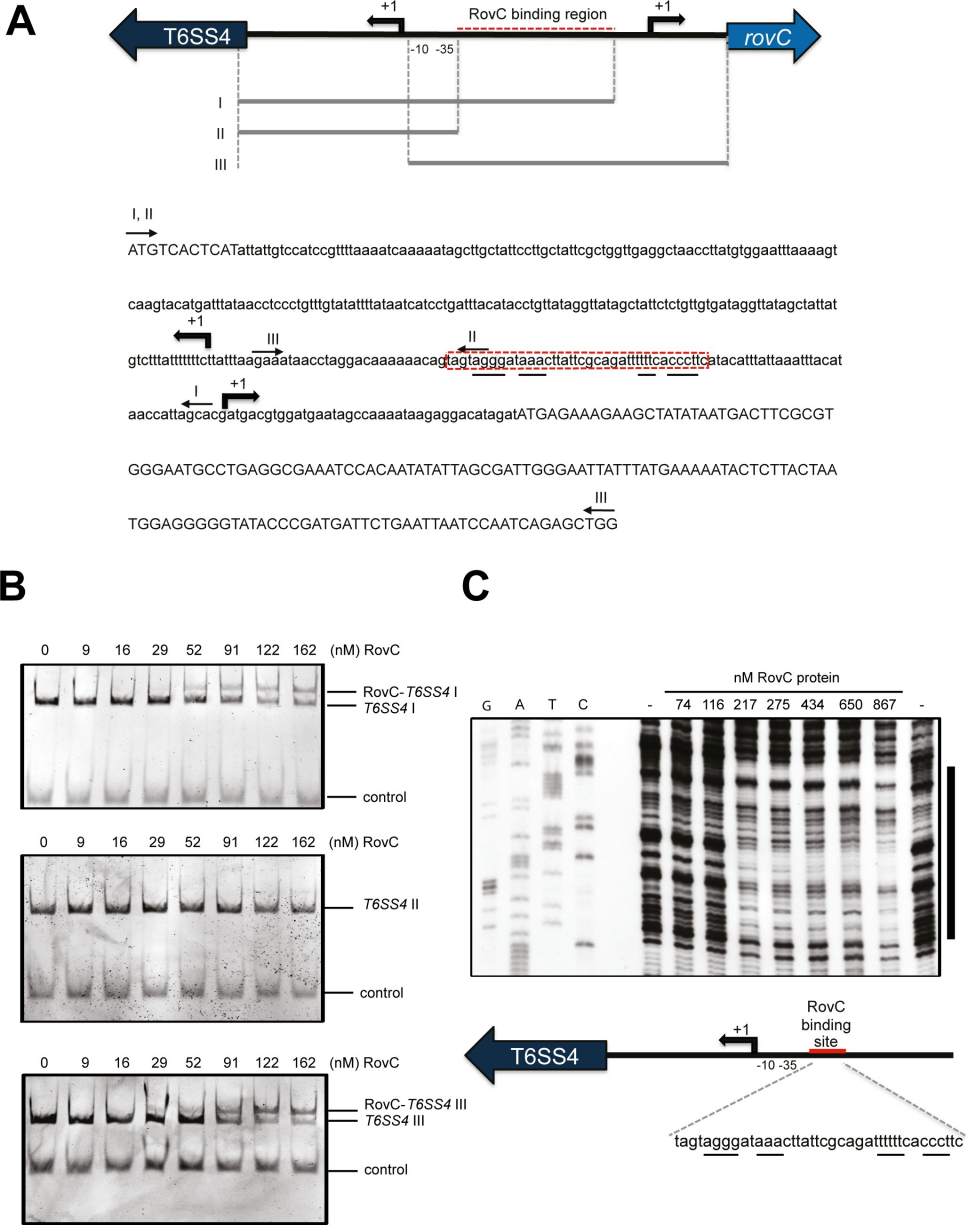
RovC specifically binds to the T6SS4 promoter region. (**A**) Schematic representation of the T6SS4 and *rovC* upstream region. The fragments harboring varying lengths of the upstream region used for the RovC-DNA binding analysis are shown below the fragment and are labeled with roman numbers (I to III). The RovC binding region is marked in red (dashed line) and the transcriptional start sites of the T6SS4 operon and the *rovC* gene are indicated with +1 and a broken arrow. The -10 and -35 regions of the T6SS4 promoter are indicated. Primers for amplification of the fragments are indicated by arrows. (**B**) DNA binding analysis with purified RovC protein. 130 fmol of DNA were incubated with increasing amounts of hexameric RovC protein (0–162 nM) for 30 min at room temperature. A *csiD* DNA fragment from *E*. *coli* was used as negative control. (**C**) DNase I footprinting assay to identify the RovC binding site using the *rovC*^+^ plasmid pSSE11 as template. The protected region is indicated on the right and is indicated in a schematic representation as a red line. RovC binds to the T6SS4 promoter in a region approximately -70 to -32 bp upstream of the T6SS4 transcriptional start site (+1). T6SS4 DNA and a sequencing reaction were amplified with a DIG-labeled primer. T6SS4 DNA was incubated with increasing concentrations of RovC protein (0–900 nM) for 30 min at room temperature and was subsequently digested with an appropriate concentration of DNase I for 30 sec. The DNA-protein samples and the sequencing reaction were separated on a 6% urea gel, transferred onto a nylon membrane and visualized with a digoxigenin antibody.

Based on this prediction, we purified RovC (**S1A Fig**) and tested whether it is able to specifically interact with the T6SS4 promoter to activate its expression. For this purpose, DNA fragments (I, II, III) harboring different portions of the T6SS4 promoter region (**Fig 3A**) were incubated with increasing concentrations of the purified RovC protein. As shown in **Fig 3B**, RovC was able to specifically bind and form RovC-DNA complexes with DNA fragments harboring the intergenic region between the transcriptional start sites of the T6SS4 operon and the *rovC* gene. To determine the precise binding sequence, we performed DNase I footprinting assays and found that RovC binds to a 39 bp sequence with a palindromic sequence immediately upstream of the -35 region of the T6SS4 promoter (**Fig 3C**).

### RovC is a novel hexameric DNA-binding protein

**Fig 4:**
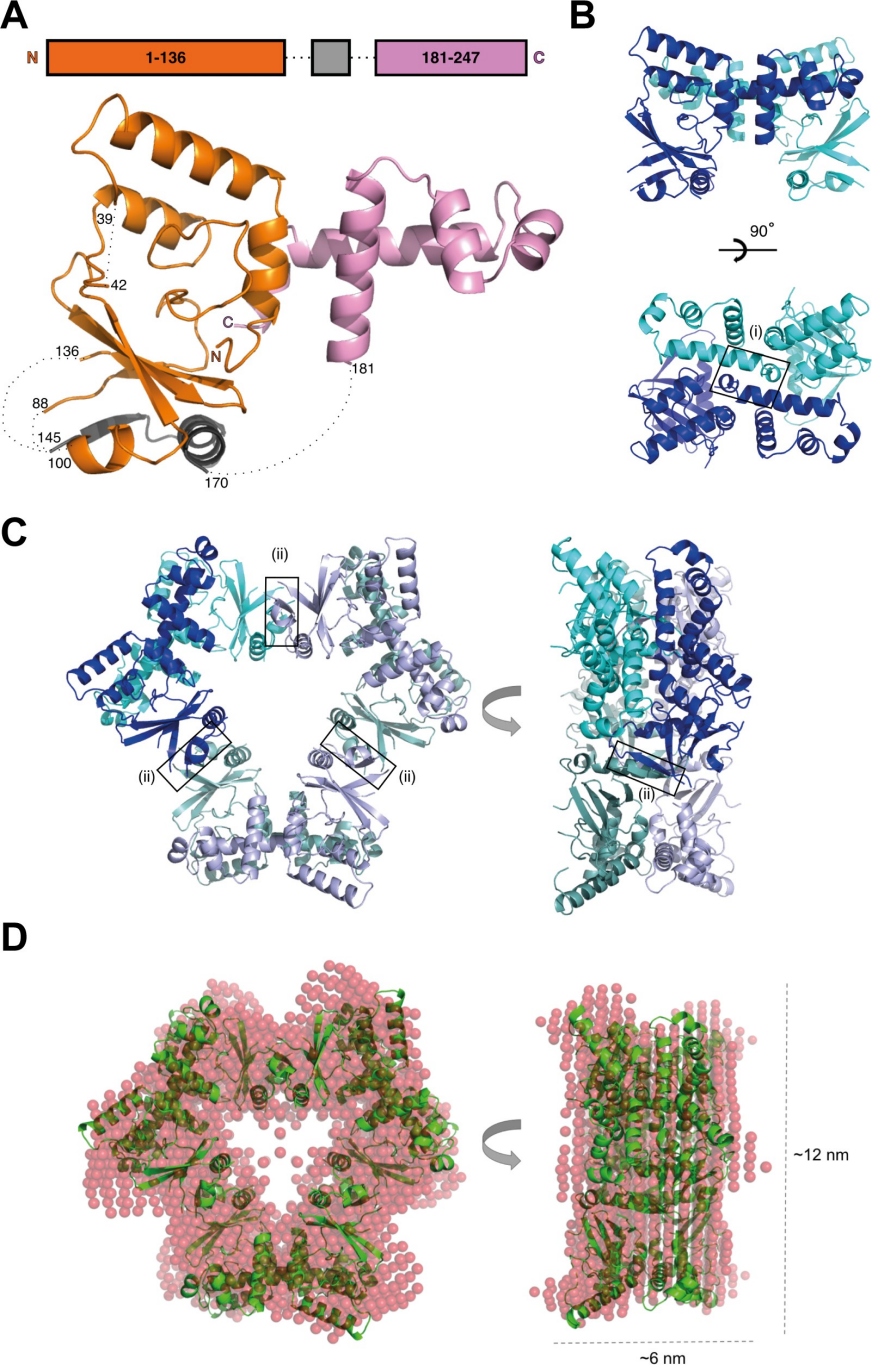
RovC forms a hexameric ring-shaped protein. (**A**) Crystal structure of RovC with domain boundaries (top) and cartoon representation of RovC (below). The N-terminal domain is illustrated in orange and gray, and the C-terminal domain in pink. An elongated stretch of amino acids (gray) folds into a secondary structure, which we can confidently assign to the central region (aa 137–180) of RovC. An unambiguous assignment of individual amino acids to this section was however not possible due to ill-defined electron density. This stretch has been assigned to position 145–170 based on secondary structure prediction. Flexible/unstructured loops are shown as black dotted lines. (**B**) Structure of the RovC dimer subunit in two orientations. The two protomers are shown in blue and cyan. The box depicts the position of the mutant A237E/G242E in interface (i). (**C**) Hexameric ring of RovC in two orientations. The hexameric ring has been generated by applying crystallographic symmetry on the monomeric RovC structure and is composed of three RovC dimers (colors of one dimer as in **B**, the other two RovC dimers are shown in darker tones of blue and green). The boxes depict the position of the mutants I150P and I150P/Y151P in interface (ii). (**D**) SAXS modelling. Individual *ab initio* model derived from DAMMIN (red spheres) spatially superimposed with the hexameric RovC-ring derived from the X-ray crystal structure (green). Orientations are as in **C**.

To gain a better insight into the structure and function of this novel DNA-binding protein, we determined its crystal structure to 2.3 Å resolution (**Fig 4A**, **S1B Fig, S1 Table**). RovC consists of two distinct domains. The C-terminal domain (181–247, pink) is formed by five α-helices which fold into a helix-turn-helix motif, as predicted. The N-terminal domain comprises amino acids 1–136 (orange) and a central region (gray). Amino acids 1–136 fold into four α-helices and a mixed β-sheet with four β-strands. While a secondary structure can be assigned to the central region (gray) that forms an elongated stretch of amino acids, an unambiguous assignment of individual amino acids to this elongated stretch (gray) was not possible due to ill-defined electron density and flexible/unstructured loops which connect it to the rest of the protein. But despite the ill-defined density in the central region, we could confidently assign this stretch to the N-terminal domain of RovC.

Size exclusion chromatography followed by multi-angle laser light scattering (SEC-MALS) further revealed that RovC assembles into a stable complex with a molecular weight of approx. 173 kDa, corresponding to a RovC-hexamer in solution (**S1C Fig, S2 Table**). The asymmetric unit of crystallized RovC contains one molecule. However, extending the crystal lattice by generating the crystallographic symmetry-related RovC molecules reveals a hexameric ring-shaped structure composed of three RovC dimers (interface between monomers: interface I) in which the interface between the dimers (interface II) is formed solely by the N-terminal domain (**Fig 4B and 4C, S3 Fig**). The hexameric ring-shaped assembly is maintained in solution, confirmed by small angle X-ray scattering (SAXS; see Supplementary Information and the Small Angle Scattering Biological Data Bank, SASBDB [37], entry SASDHP5) (**Fig 4D, S2 and S3 Tables**).

To confirm the multimeric structure and to characterize the DNA-binding mode of the protein, we performed a structure-guided mutational analysis of RovC. Mutations were introduced that (i) disrupt the identified dimeric interface (A237E, G242E in the dimer interface region of residues 231–246), (ii) disrupt the ring-shaped hexamer (I150P, I150P/Y151P in the 147–151 β-strand forming the interface in the ring-shaped hexamer) and (iii) are located on the outer surface of the hexameric ring and are not supposed to destabilize the ring-shaped hexamer (S219E/A220E) (**Fig 4B and 4C, S3 Fig**). To analyze the effect of these mutations on the oligomeric state, SEC-MALS was performed. Molecular mass calculation showed that the RovC S219E/A220E mutant forms a soluble and stable hexamer (169 kDa), whereas the RovC I150P, I150P/Y151P, A237E and G242E mutant proteins resulted in insoluble proteins or unstable hexamers, further validating the ring-shaped model of RovC (**S2 Table**).

**Fig 5:**
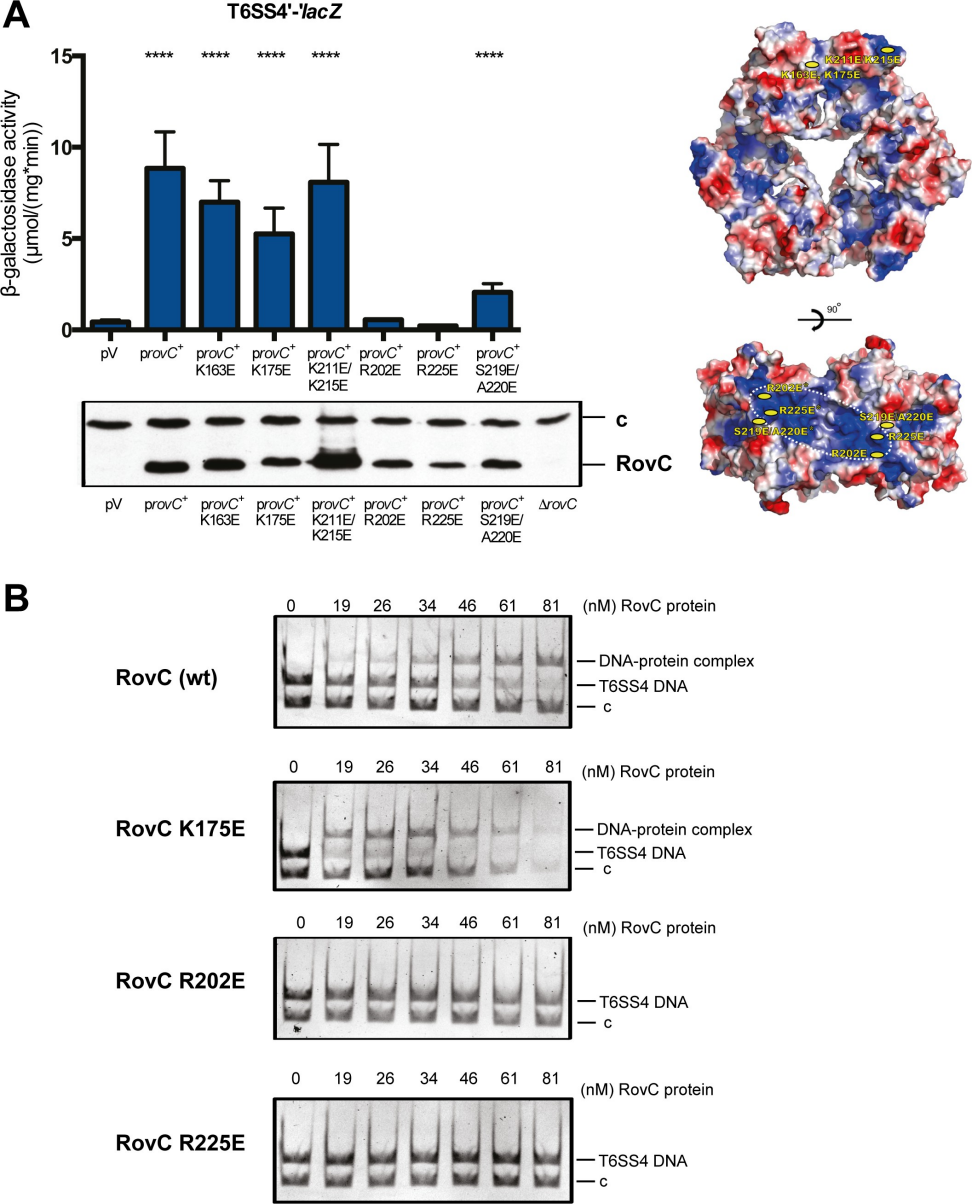
DNA-binding of RovC is required for induction of the T6SS4 operon. (**A**) Expression of a translational T6SS4’-‘*lacZ* reporter fusion (pSSE64) was measured in *Y*. *pseudotuberculosis* wild-type strain YPIII. YPIII was transformed with the empty vector pZA24 (pV) and complemented with the arabinose-inducible *rovC*^+^ overexpression plasmids pVK46 (p*rovC*^+^), pVK47 (p*rovC*^+^ K163E), pVK48 (p*rovC*^+^ K175E), pVK49 (p*rovC*^+^ K211E/K215E), pVK50 (p*rovC*^+^ R202E), pVK51 (p*rovC*^+^ R225E) and pVK52 (p*rovC*^+^ S219E/A220E). Strains were grown over night for 12 h at 25°C in LB medium. Expression of the P_*ara*_ promoter was induced by addition of 0.1% arabinose and β-galactosidase activity (μmol/mg^.^min) was measured after 4 h (left upper panel). The data represent the mean ± standard deviation of two independent experiments, carried out in triplicates. Data were analyzed by the Student’s t-test; **** P < 0.0001, *** P < 0.001; n.s.: not significant) (top). Western blot to monitor the amount of synthesized RovC protein. Whole cell extracts were prepared from overnight cultures grown for 16 h at 25°C in LB medium, separated on 15% SDS gels and transferred onto a PVDF membrane. Proteins were detected by immunoblotting with a polyclonal antibody directed against RovC. An unspecifically detected protein band was used as loading control (c) and the Δ*rovC* mutant strain served as negative control (left lower panel). Electropotential surface analysis of RovC hexamer is shown on the right (right panel) in two orientations. Potentials between -69.553 kT/e (red) and + 69.553 kT/e (blue) are color-coded. The positions of mutants used in the *in vivo* induction analysis (**A**, left panel) and *in vitro* DNA-binding analysis by EMSA (**B**) are marked. (**B**) EMSAs with purified wild-type and different mutant variants (RovC K175E, RovC R202E and RovC R225E). 130 fmol of DNA were incubated with increasing amounts of the different hexameric RovC proteins (0–81 nM) for 30 min at room temperature. A *csiD* DNA fragment (c) from *E*. *coli* was used as negative control.

To identify the DNA-binding domain of RovC, the electrostatic potential, calculated by Pymol was plotted on the surface of the RovC hexamer [38]. One extended basic patch (formed by a RovC dimer) was identified on the outer surface of the ring mainly formed by residues 225–240 present on the helix-turn-helix motif which is likely to be involved in the interaction with the negatively charged DNA backbone (**Fig 5A**). A comparative sequence analysis identified hypothetical proteins of different Gram-negative bacteria in the NCBI database that share ≥44% sequence identity with RovC (**S4 Fig**). In particular, the C-terminal region (residues 219–240 of RovC) that overlaps with the basic patch is highly conserved, giving rise to the notion that these proteins are likely to adopt a similar fold and possess comparable DNA-binding function.

To study the role of the extended basic patch for DNA-binding, site-directed mutagenesis of the basic and solvent-exposed residues of this region (R225E, R202E), residues in the vicinity (S219E/A220E) or outside this region (K175E, K163E, K211E/K215E) was performed. The resulting mutant proteins were expressed in *Y*. *pseudotuberculosis* YPIII and their ability to induce the expression of the translational T6SS4’-‘*lacZ* fusion was tested (**Fig 5A**). All RovC variants with amino acid substitutions within or close to the basic patch were unable to induce expression of the T6SS4’-‘*lacZ* fusion. To confirm this result, we purified the non-inducing variants R225E, R202E and the inducing K175E as positive control to test their DNA-binding ability. Prior to examining the DNA binding activity of mutants, MALS was performed to ensure that the R225E and R202E mutations did not affect the hexameric state of the protein. Both non-inducing mutants form hexamers in solution (**S2 Table**). DNA binding assays further revealed that the R225E and R202E substitution completely abolished the DNA-binding activity of RovC, while the DNA-binding activity of the inducing K175E mutant remained unaffected (**Fig 5B**). This strongly indicated that the DNA-binding region resides within the conserved basic patch of RovC.

### Analysis of *rovC* expression

Previous results in this study demonstrated that expression of *rovC* is tightly controlled and repressed under standard growth conditions. As environmental parameters, such as temperature changes and nutrient depletion are crucial signals in the control of *Yersinia* virulence gene expression, we first asked whether changes in these parameters influence *rovC* mRNA levels. Expression analysis of a translational *rovC’-‘lacZ* fusion and northern blot experiments using a *rovC* specific probe revealed that *rovC* expression is mostly favored at 25°C during stationary phase (**S5A and S5B Fig**). This indicates that observed upregulation of the T6SS4 transcription under these conditions (**S5C Fig**; [17, 19]) occurs through upregulation of RovC. As negative feedback-loops are often used as control elements to limit unfavored expression of transcriptional regulators, we also monitored whether RovC is able to control its own transcription. For this, the activity of a *rovC'*-*'lacZ* fusion was assessed in the *Y*. *pseudotuberculosis* wildtype and the isogenic Δ*rovC* mutant, but no difference was observed, demonstrating that *rovC* is not autoregulated on the transcriptional level (**S5D Fig**).

### Dual-level control of *rovC* transcription and transcript stability by CsrA

As the RovC activator protein was identified as a factor that is strongly upregulated in the absence of CsrA (**Fig 1B**), we further investigated how *rovC* expression is influenced by and linked with the Csr system and other regulators (e.g. RovM), which are part of the Csr regulatory network in *Y*. *pseudotuberculosis*.

**Fig 6:**
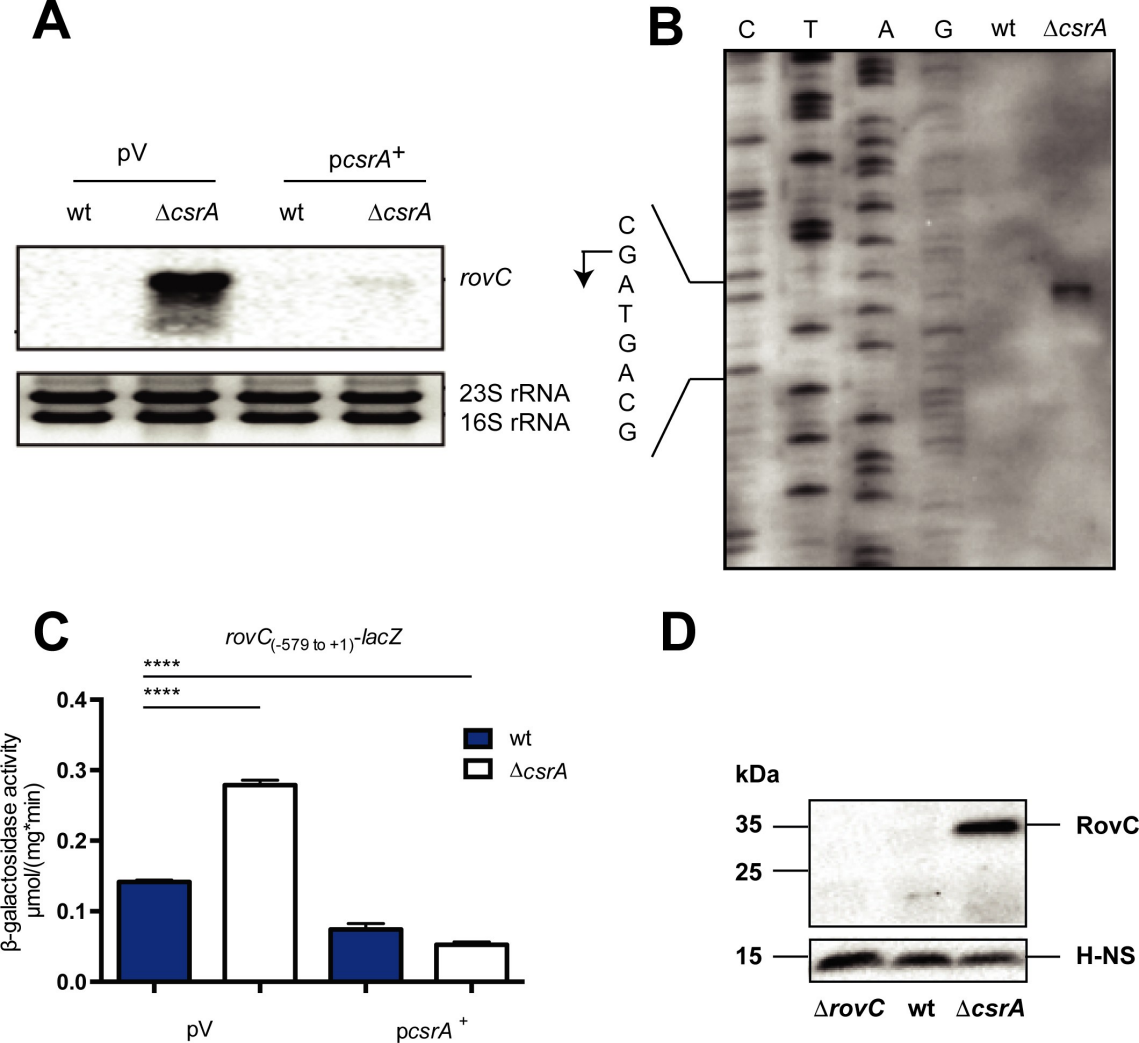
CsrA represses transcription of *rovC*. (**A**) Comparison of the *rovC* mRNA transcript levels in the *Y*. *pseudotuberculosis* wild-type (YPIII) and the *csrA* mutant strain (YP53) by northern blotting. Both strains were transformed with the empty vector pAKH85 (pV) or with the overexpression *csrA*^+^ plasmid pAKH56 (p*csrA*^+^). Strains were grown in LB medium at 25°C to stationary phase. Total RNA was prepared, separated on 0.7% MOPS agarose gels, transferred to a nylon-membrane and probed with a digoxigenin (DIG)-labeled *rovC* fragment. 16S and 23S rRNAs were used as loading controls. (**B**) Primer extension to determine the transcriptional start site of *rovC*. RNA was prepared from the *Y*. *pseudotuberculosis* strain YPIII and the Δ*csrA* mutant strain. 20 μg of RNA and a DIG-labeled primer specific for the *rovC* coding region were used for the primer extension. The sequencing reaction amplified with the identical *rovC* primer is shown on the left. The *rovC* transcription start site is indicated by an arrow and the nucleotide sequence of the TSS is given on the left. (**C**) Expression of a transcriptional *rovC*-*lacZ* reporter fusion (pAKH189) was monitored in the YPIII and in the Δ*csrA* strain. Both strains were transformed with the empty vector pAKH85 (pV) or the *csrA*^+^ plasmid pAKH56 (p*csrA*^+^). β-galactosidase activity (μmol/mg^.^min) was measured after strains were grown over night for 16 h at 25°C in LB medium. The data represent the mean ± standard deviation of two independent experiments, carried out in triplicates. Data were analyzed by the Student’s t test; **** P < 0.0001. (**D**) Comparative analysis of RovC protein levels. Samples were prepared from the wild-type, the Δ*csrA* and the Δ*rovC* mutant. Whole cell extracts were prepared from overnight cultures grown at 25°C in LB medium, separated on 15% polyacrylamide gels and transferred onto a PVDF membrane. Proteins were detected by immunoblotting with a polyclonal antibody directed against RovC or H-NS.

First, we examined the influence of CsrA on the abundance of the *rovC* mRNA. Northern blot and primer extension analysis demonstrated that *rovC* expression is repressed in the presence of the CsrA protein, i.e. *rovC* transcripts were not detectable in the wildtype. In contrast, high *rovC* mRNA levels were monitored in the Δ*csrA* mutant strain, whereby introduction of a *csrA*^+^ plasmid restored repression of *rovC* (**Fig 6A and 6B**). To define the underlying molecular mechanism, we first tested the influence of CsrA on *rovC* transcription. For this purpose, we performed a primer extension analysis and determined the transcriptional start site at position -39 nt relative to the translational start (**Fig 6B**). Next, we tested a *rovC-lacZ* reporter fusion harboring the *rovC* regulatory region to position -579 relative to the identified transcriptional start site and found the *rovC* was significantly more expressed in the absence of CsrA, and this effect could be also complemented by a *csrA*^+^ plasmid (**Fig 6C**). To verify this result, we tested expression of *rovC* and were able to detect significant amounts of the RovC protein in the Δ*csrA* mutant, but no protein was observed in the wildtype strain (**Fig 6D**). In summary, this demonstrated that CsrA inhibits *rovC* transcription. As CsrA is a post-transcriptional RNA-binding regulator, observed CsrA-dependent repression of *rovC* transcription seems to be indirect.

**Fig 7:**
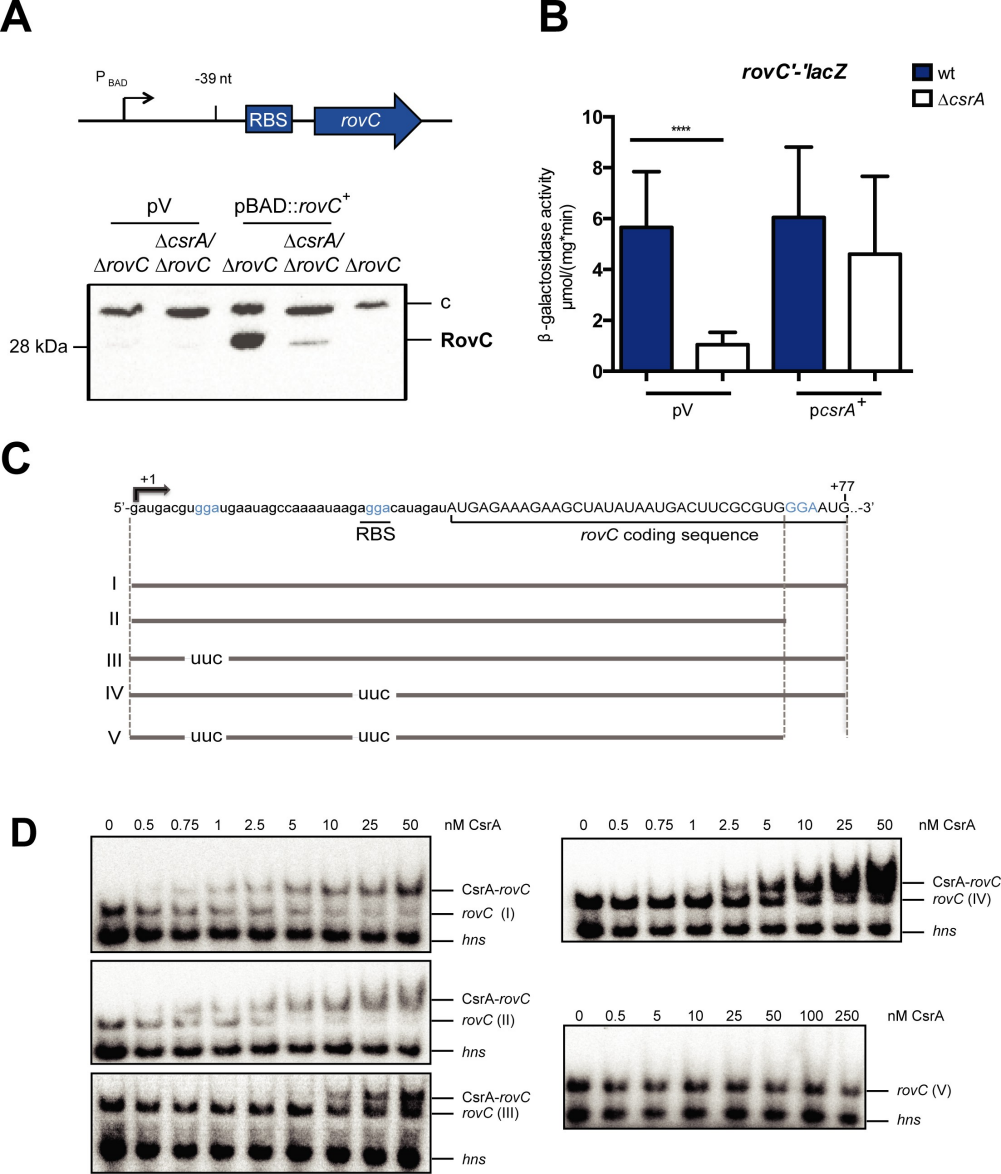
CsrA directly interacts with the *rovC* transcript. (**A**) RovC protein amounts were monitored in the *Y*. *pseudotuberculosis* Δ*rovC* strain (YP154) and the Δ*csrA/*Δ*rovC* double mutant (YP318). Both strains were transformed with the empty vector pBAD30 (pV) or the inducible *rovC*^+^ overexpression plasmid pVK25 (pBAD::*rovC*^+^). The promoter was induced by addition of 0.1% arabinose. Whole cell extracts were prepared from strains grown overnight at 25°C, separated on 15% SDS gels and transferred onto a PVDF membrane. Proteins were detected by immunoblotting using a polyclonal RovC antibody. The Δ*rovC* mutant strain served as negative control and a detected unspecific band was used as loading control. (**B**) Expression of a translational *rovC*’-‘*lacZ* reporter fusion (pVK43) was monitored in the *Y*. *pseudotuberculosis* wild type strain (YPIII) and the Δ*csrA* mutant (YP53). Both strains were transformed with the empty vector pAKH85 (pV) or complemented with the *csrA*^+^ plasmid pAKH56 (p*csrA*^+^). β-galactosidase activity (μmol/mg^.^min) was measured in strains grown over night at 25°C. The data represent the mean ± standard deviation of three independent experiments, carried out in triplicates. Data were analyzed by Student’s t-test; ****P < 0.0001. (**C**) Schematic representation of the *in vitro* transcribed *rovC* mRNA fragments. The three GGA-motifs within the RNA fragments are highlighted in blue, the transcriptional start site is labeled with +1 and the end of the fragment with +77. The RBS and start of the *rovC* coding sequence are underlined. Fragments containing varying length of the *rovC* mRNA are labeled with roman numerals ranging from I to V. All substitutions of GGA-motifs within the fragments are indicated as ‘uuc’. The fragments range from +1 to +77 or from +1 to +71; they are depicted by grey lines below the *rovC* mRNA sequence. (**D**) EMSAs with RNA fragments I to V show direct binding of the CsrA protein to the *rovC* transcript. The *hns* transcript was used as negative control. The RNAs were incubated with increasing concentrations of purified CsrA ranging from 0 to 50 nM (I—IV) or 0 to 250 nM (V).

The strong influence of CsrA on *rovC* transcript and RovC protein levels prompted us to determine whether CsrA also influences RovC synthesis directly on a post-transcriptional level. For this purpose, the *rovC* gene was expressed under the control of the P_BAD_ promoter (P_BAD_::*rovC*^+^) to exclude CsrA influence on *rovC* transcription in the Δ*rovC* or the Δ*rovC/*Δ*csrA* mutant. Surprisingly, our western blot analysis revealed that the amount of the RovC protein is significantly reduced in absence of CsrA (**Fig 7A**). This influence was confirmed by monitoring the expression of a translational *rovC'-'lacZ* fusion harboring the complete *rovC* 5'-UTR (-39 to +1 relative to the transcriptional start site) and the first 17 amino acids of *rovC* fused to the *lacZ* gene under the control of the P_BAD_ promoter (**Fig 7B**). This indicated that CsrA, which mainly represses RovC synthesis on the transcriptional level, counteracts this activity to some extent on the post-transcriptional level.

To determine whether the positive effect on RovC synthesis is direct and occurs through the interaction of the CsrA molecule with the *rovC* transcript, we first inspected the *rovC* transcript for potential CsrA binding sites. CsrA homodimers preferentially interact with GGA motifs in single-stranded loop-regions of hairpin structures. Most CsrA RNA targets harbor two GGA-containing stem-loops that are separated by 10–63 nucleotides in the 5’-untranslated region (5’-UTR) [39, 40, 41, 42]. Two potential GGA-containing CsrA binding motifs were found within the 5-'UTR of *rovC*. One GGA motif is located close to the transcriptional start site (TSS) (+ 9 nt relative to the TSS), a second GGA motif is located within the ribosomal binding site (RBS) (+ 30 nt relative to the TSS). A third putative binding motif is located in the coding region (+ 32 nt relative to the start codon) (**Fig 7C**).

To test the interaction of CsrA to the different potential binding sites, RNA electrophoretic mobility shift assays (EMSAs) were performed with purified CsrA and *rovC* transcript fragments containing all three motifs as well as deleted or mutated versions of the GGA motifs (**Fig 7C and 7D**). The assays revealed that CsrA exhibits its highest binding affinity to the *rovC* mRNA fragments harboring the first two GGA motifs; the last motif is unnecessary for binding. The *rovC* mRNA fragments, in which one or two of the GGA motifs were mutated to UUC, required considerably higher protein concentrations for CsrA-*rovC* complex formation, whereas no CsrA binding was observed in the absence of all three GGA motifs (**Fig 7D**).

**Fig 8:**
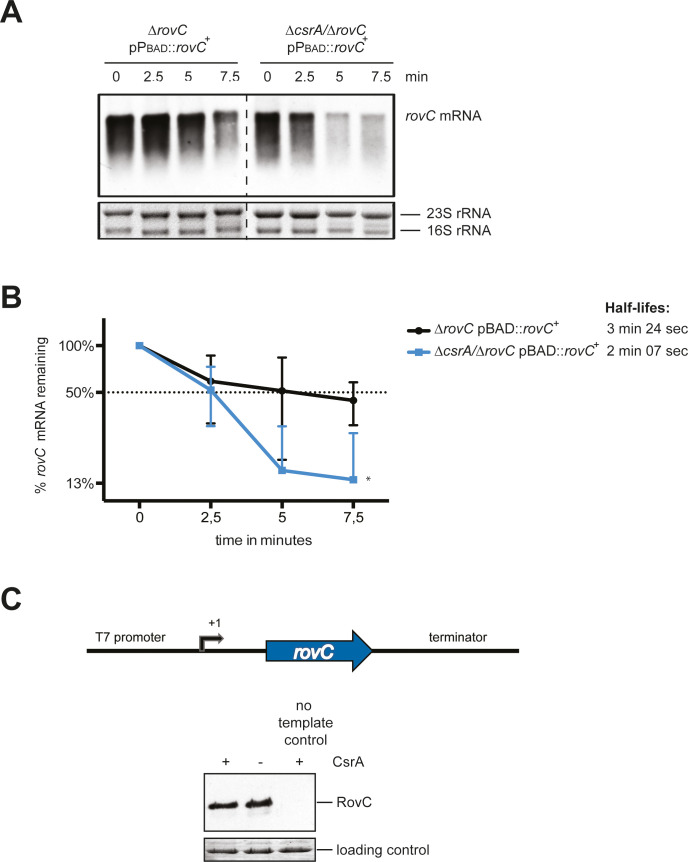
CsrA stabilizes the *rovC* mRNA transcript. RNA stability assay for comparison of the mRNA half-life in dependence of CsrA. (**A**) For this, strains Δ*rovC* (YP148) and Δ*csrA*/Δ*rovC* (YP318) were complemented with the arabinose inducible *rovC*^+^ overexpression plasmid pVK25 (pP_BAD_::*rovC*^+^). Cultures were grown to exponential phase at 25°C, and the P_BAD_ promoter was induced by addition of 0.1% arabinose. Transcription was stopped by addition of rifampicin, and samples were taken directly after (0 min) 2.5, 5 or 7.5 minutes. Total RNA was isolated, separated on a 1.2% MOPS gel, transferred onto a nylon membrane and probed with a DIG-labeled *rovC* encoding PCR-fragment. 16S and 23S rRNAs were used as loading control. One representative blot is shown. (**B**) The relative band intensity of the northern blots was calculated in relation to the 16S and 23S rRNAs. The graph represents the remaining percentage (y-axis) of *rovC* mRNA over time (x-axis) on a half-logarithmic scale. The half-life of the *rovC* mRNA was calculated via exponential regression from three independent experiments. Asterisks indicate results which differ significantly from each other; *P < 0.05. (**C**) Schematic representation of the *rovC* DNA fragment used for the *in vitro* transcription-translation assay. The fragment harbors a T7 promoter, the complete 5’-UTR (-39 to +1 relative to the transcriptional start site), the coding sequence of the *rovC* gene and a terminator sequence (top). To analyze the effect of CsrA on *rovC* translation, an *in vitro* transcription-translation assay was performed with and without 100 nM CsrA protein. As negative control, a reaction mixture without template was incubated with 100 nM CsrA. Subsequently, the samples were separated on a 15% SDS gel and transferred to a PVDF membrane. RovC protein was detected using a polyclonal RovC antibody. Ponceau S Red stained protein bands were used as loading control (bottom).

As CsrA binding to a target mRNA often also affects the stability of the transcript [28, 43], we tested whether CsrA-binding to the *rovC* mRNA influences the degradation of the *rovC* transcript. To do so, synthesis of the *rovC* transcript was induced under the control the P_BAD_ promoter in the Δ*rovC* and the Δ*csrA/*Δ*rovC* mutant to exclude transcriptional effects. Transcription was stopped by addition of rifampicin and *rovC* mRNA levels were quantified after different time points. As shown in **Fig 8A and 8B**, the *rovC* mRNA was more rapidly degraded in the absence of CsrA, indicating that CsrA binding to the *rovC* transcript seems to stabilize the *rovC* transcript to a certain extent. We further examined whether CsrA also affects the translation efficiency of *rovC*. For this purpose, an *in vitro* transcription-translation assay was performed in the presence and absence of CsrA (**Fig 8C**). Similar amounts of *in vitro* synthesized RovC protein were observed with and without CsrA, indicating that this riboregulator does not modulate the translation efficiency of the *rovC* mRNA.

### RovM-promoted activation of T6SS4 expression requires RovC

**Fig 9:**
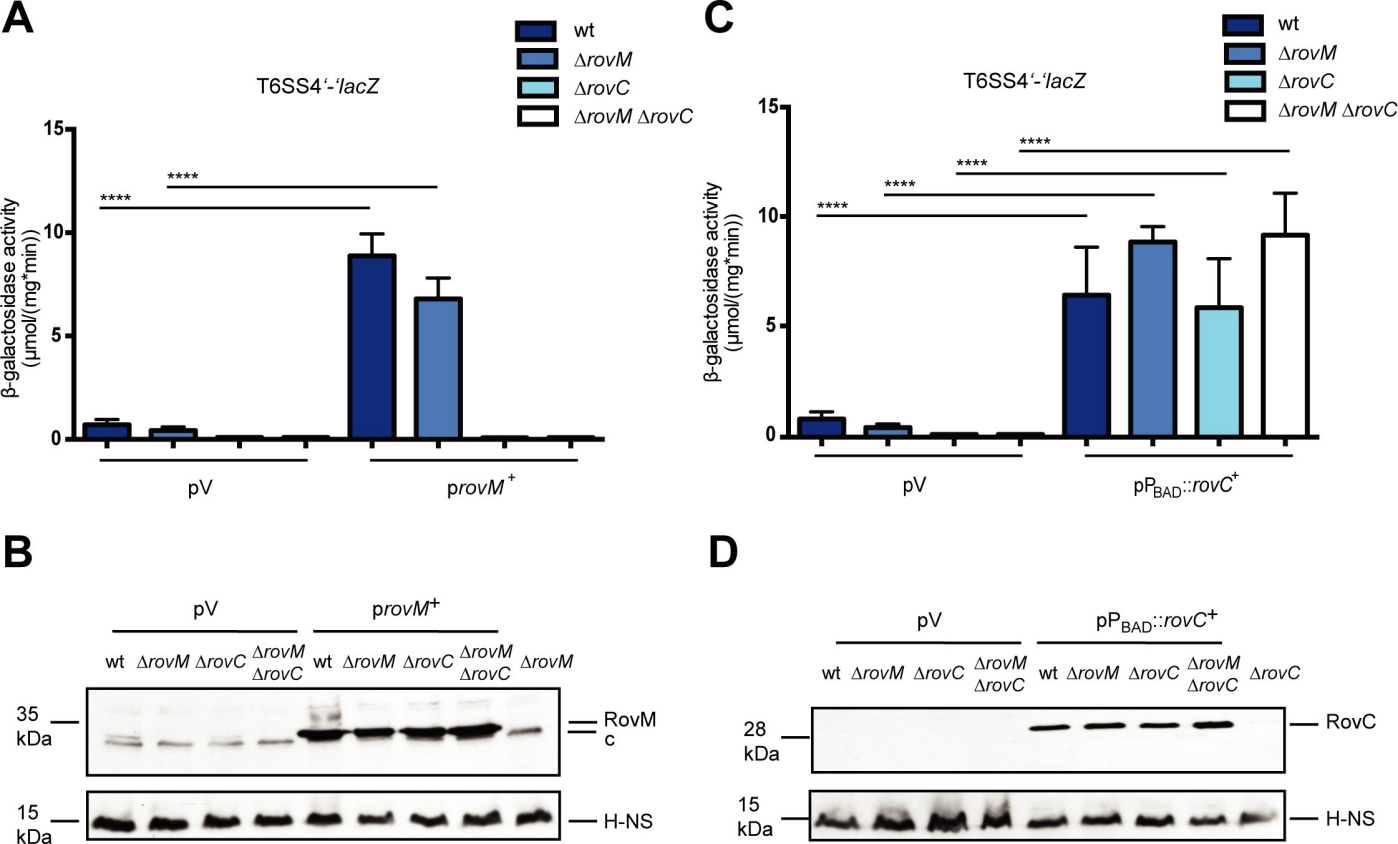
RovM-dependent T6SS4 activation. Expression of a translational T6SS4’-‘*lacZ* fusion (pSSE64) was analyzed in the *Y*. *pseudotuberculosis* wild type strain YPIII, the Δ*rovM* (YP72), the Δ*rovC* (YP154) and the Δ*rovC/*Δ*rovM* mutant (YP338). The strains were transformed with (**A**) the empty vector pV (pIV2) or were complemented with the *rovM*^+^ overexpression plasmid pAKH64 (p*rovM*^+^) or (**C**) transformed with the empty vector pZA24 (pV) or complemented with the inducible *rovC*^+^ plasmid pVK46 (pP_ara_::*rovC*^+^). β-galactosidase activity (μmol/mg^.^min) was measured after strains were grown over night at 25°C. The data represent the mean ± standard deviation of two independent experiments, carried out in triplicates. Data were analyzed by the Student’s t test; ** P<0.01. RovM (**B**) or RovC (**D**) protein amounts were monitored in the wild type strain (YPIII), the Δ*rovM* (YP72), the Δ*rovC* (YP154) and the Δ*rovC*Δ*rovM* mutant (YP338), transformed with (**B**) the empty vector pIV2 (pV) or the *rovM*^+^ overexpression plasmid pAKH64 (p*rovM*^+^) or (**D**) the empty vector pZA24 (pV) or the *rovC*^+^ overexpression plasmid pVK46 (pP_ara_::*rovC*^+^). Whole cell extracts were prepared from cultures grown overnight at 25°C, separated on 15% SDS gels and transferred onto a PVDF membrane. Proteins were detected by immunoblotting with a polyclonal RovM (**B**) or RovC (**D**) antibody and H-NS protein amounts were used as loading control. The respective mutant strains served as negative controls.

Previous studies demonstrated that the transcriptional LysR-type virulence regulator RovM, which depends on available nutrients and the Csr system [24, 27], interacts directly with the intergenic region upstream of P_T6SS4_ (**Fig 1A**), and activates expression of the T6SS4 operon [26]. This implied that RovM and RovC could impact or complement each other with regard to T6SS4 activation. To investigate this issue, we first compared the expression of the T6SS4’-‘*lacZ* fusion between wildtype, the Δ*rovM* and the Δ*rovC* mutant (**Fig 9A and 9B**). Expression of the T6SS4’-‘*lacZ* fusion was not significantly decreased in the *rovM*-deficient strain, but it increased strongly (10-fold) upon overexpression of RovM (**Fig 9A**). In contrast, T6SS4 expression in the Δ*rovC* and Δ*rovC/*Δ*rovM* mutant remained strongly repressed, even when *rovM* was overexpressed. As neither loss nor overexpression of RovM affected expression of the *rovC’*-‘*lacZ* reporter (**S6 Fig**), we assume that RovC is required for RovM-promoted activation, but this does not involve a RovM-dependent induction of *rovC* expression.

We further monitored T6SS4’-‘*lacZ* activity in the *Y*. *pseudotuberculosis* wildtype and the Δ*rovM*, Δ*rovC* and Δ*rovM/ΔrovC* mutant strains transformed with a *rovC*^+^ plasmid (**Fig 9C and 9D**). Complementation with RovC resulted in a strong induction of the T6SS4’-‘*lacZ* fusion in all tested strains. The detection of equal amounts of RovC (**Fig 9D**) and of equal expression levels of the *rovC’-‘lacZ* fusion (**S6 Fig**) in the *rovM*-deficient mutants shows that RovM has no influence on RovC synthesis. Based on these results we assume that RovC is an essential activator of the T6SS4 operon, which can fully compensate the loss of RovM when expressed.

## Discussion

Bacterial species can carry several T6SSs, which allow them to use a plethora of different effector proteins and toxins to subvert host cells or attack prey cells. Most T6SSs, including those of human pathogenic *Yersinia* species, are repressed under laboratory growth conditions. This strongly suggests that host cell contact/tissue colonization, presence of competitors or certain environmental factors are required to trigger their expression and assembly.

In this study, we identified a novel transcriptional activator—RovC—of *Yersinia*, which is essential for T6SS4 gene induction. RovC is highly conserved among *Y*. *pestis* (upstream of the T6SS-A cluster in opposite orientation, [16]) and *Y*. *pseudotuberculosis* strains (upstream of the T6SS4 cluster in opposite orientation, [16]) and was identified in *Y*. *pekkanenii* (80% identity) found in soil and on lettuce. Sequences of homologous proteins with so far unknown function can be found in other mammalian, plant and insect pathogenic bacteria, including *Serratia*, *Trabusiella*, *Enterobacillus*, and *Erwinia* species (**S4A Fig**). Notably, all of them encode a related T6SS and in four of the species the *rovC* homologous gene is encoded in close proximity to the T6SS cluster, indicating a broader conserved gene activation mechanism.

We show that RovC forms a new type of hexameric, ring-shaped DNA-binding protein, which interacts specifically with an extended 39 bp segment closely upstream of the promoter region of the T6SS4 gene cluster. A mutant analysis further demonstrated that nucleic acid binding occurs at the surface of the RovC hexamer, most likely by the exposed C-terminal helix-turn-helix like motif. This implies that the DNA is wrapped around the protein, unlike classic dimeric repressors, which typically surround the DNA double helix [44]. In addition, an extended basic patch was found on the surface of the RovC dimer, which might also be involved in the interaction with the negatively charged DNA backbone. In fact, non-DNA binding RovC mutant proteins, such as RovC R202E, and RovC R225E with exchanges within the basic patch (**Fig 5**) and short palindromic sequences in the operator region imply that specific base contacts are involved in the individual RovC-DNA contact. The protected operator site further indicates that not all subunits, but only 2–4 monomers are involved in DNA-binding.

Up to date, only a few hexameric nucleic acid-binding proteins are known, but their overall structures show no obvious similarity with the RovC-type hexamer. Among them are helicases and replication initiator proteins participating in the replication process [45, 46, 47]. Recent studies revealed that the dsDNA wraps around the hexameric ring of the double hexameric mini-chromosome-maintenance (MCM) helicase and the bacterial initiator DnaA. This interaction seems to require a conformational change to expose the DNA-binding motif in case of MCM, and appears to modify the degree of supercoiling to facilitate DNA melting [45, 46, 48]. A similar mechanism can also be suggested for RovC. Notably, also the multi-functional ArgR/AhrC protein binds DNA as a hexamer [49]. This transcriptional regulator is formed by arginine-mediated dimerization of identical trimers and is a direct sensor and transcriptional transducer of L-arginine concentration. It activates catabolic genes for arginine degradation and represses genes for arginine biosynthesis [50]. In addition, ArgR was found to act as an accessory factor in the resolution of plasmid ColE1 concatemers by promoting intramolecular site-specific recombination through the synapsis and activation of the XerCD recombinase [51, 52]. ArgR cooperatively interacts with four imperfect palindromic operator sites over 40 bp via only a very limited number of specific nucleic acid interactions implying distortion toward the protected major groove [49]. Another class of hexameric transcriptional regulators are the σ^54^-dependent AAA+ enhancer binding proteins, including the phage shock protein PspF, the nitrogen regulatory protein NtrC and the nitrogen fixation regulator and NifA, as well as the Zn^2+^ responsive regulator ZraR [53, 54]. These proteins consist of the N-terminal regulatory receiver domain of a two component phosphorelay system, a central catalytic AAA+ (ATPase) domain, and a C-terminal helix-turn-helix DNA-binding domain. The regulatory domain usually serves as inhibitor which is alleviated upon reception of the activation signal (e.g. phosphorylation, ligand binding). This allows DNA-binding as dimer, subsequent oligomerization to a hexamer and DNA bending whereby the AAA+ domain is brought in close to interact with σ^54^ and the RNA-polymerase to activate open complex formation and transcription coupled with ATP hydrolysis [54, 55]. The molecular mechanisms how RovC engages the extended operator site to facilitate DNA melting and transcription, and whether additional target genes exist are still unknown and are part of future studies.

**Fig 10:**
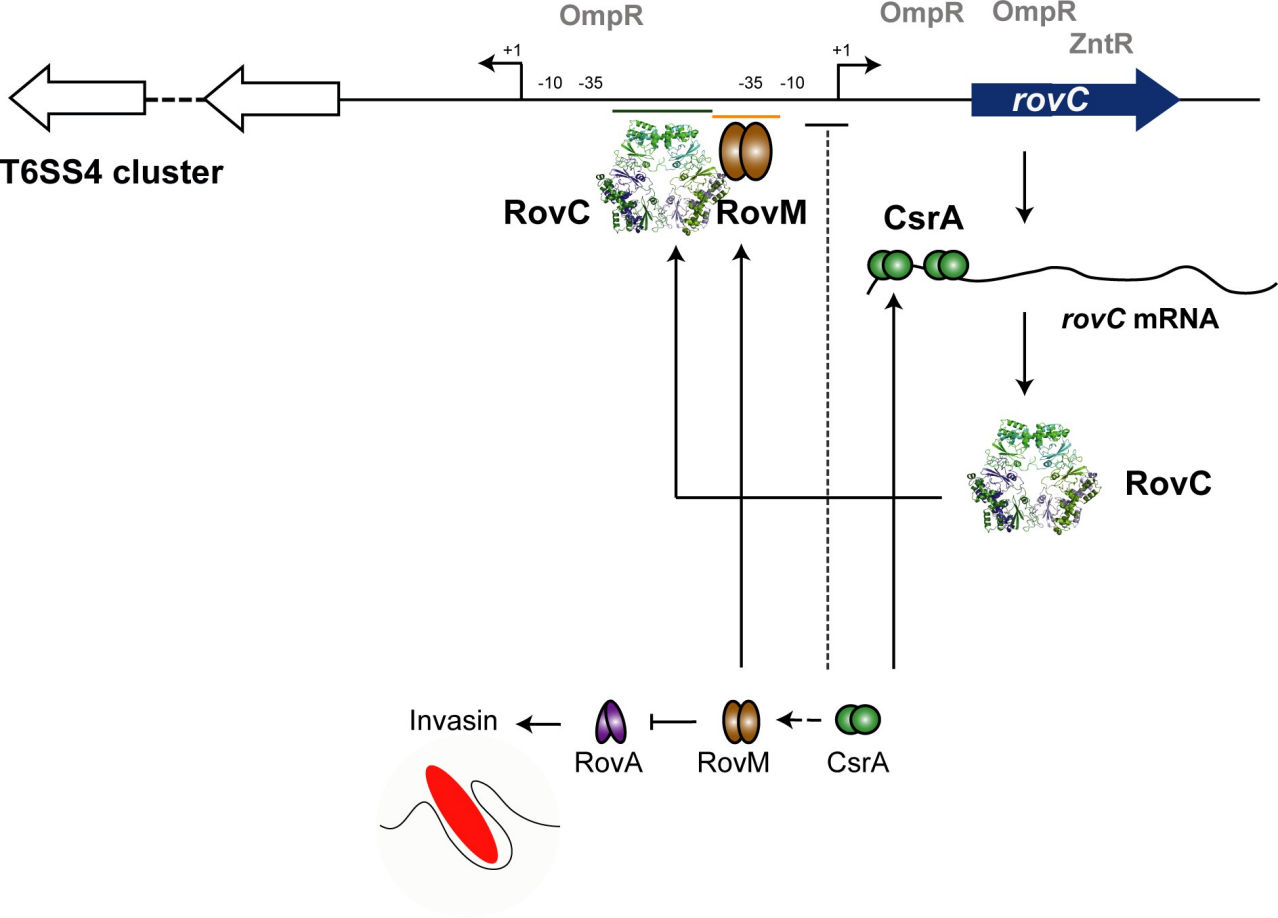
Model of *rovC* regulation and RovC-dependent T6SS4 activation. Schematic representation of the T6SS4 and *rovC* upstream region. The RovC binding region is marked in green, the RovM binding site in yellow. The transcriptional start sites of the T6SS4 operon and the *rovC* gene are indicated with +1 and a broken arrow. The -10 and -35 regions of the T6SS4 and the *rovC* promoter are indicated. The hexameric regulatory factor RovC is illustrated with its crystal structure, the dimeric regulators CsrA, RovM and RovA are indicated by symbols. Activation of gene expression is illustrated by arrows; indirect inhibition of expression is given by a dashed line.

The regulatory events triggering *rovC* gene expression and the RovC-induced T6SS4 gene cluster are many-fold and involve the global carbon source-responsive regulator CsrA, acting on the post-transcriptional and transcriptional level (**Fig 10**). CsrA strongly represses the synthesis of RovC and the T6SS4 machinery under non-inducing conditions. Environmental cues, which lead to an alleviation of this process are likely instrumental to activate *rovC* expression. The regulatory network controlling this event is highly complex and includes the LysR-type regulator RovM and most probably other regulatory factors previously shown to control T6SS4 expression [17, 19, 20, 21, 22, 23, 26].

CsrA, which integrates disparate signals into global responses is upstream of the RovM-RovA-InvA/PsaA regulatory cascade, regulating expression of the primary invasion factor invasin (InvA) [27], and it is implicated in the control of the virulence plasmid-encoded Ysc/Yop T3SS [56, 57]. CsrA of *Yersinia* absorbs multiple sensory information and acts as modulator to continuously adapt its biological fitness and virulence-relevant phenotypic responses [29]. The function of CsrA is controlled by the small RNAs CsrB and CsrC, which sequester CsrA and this would alleviate *rovC* repression. CsrB/CsrC levels are controlled by two two-component systems, the BarA/UvrY controlling *csrB* and the PhoP/PhoQ system, which influences *csrC* directly [27, 30, 58]. This regulatory pathway could allow the bacteria to control T6SS4 expression in response to various different carbon sources/nutrients as well as antimicrobial peptides, low pH, and ions such as Mg^2+^ which are sensed by the two-component systems. Moreover, CsrA seems to promote reciprocal regulation of the *Yersinia* T6SS4 and Ysc-T3SS; the latter was recently shown to be activated by CsrA [59].

Notably, influence of the Csr system is not restricted to the T6SS4 system of *Y*. *pseudotuberculosis*. Recent studies demonstrated that the CsrA homolog RsmA negatively controls all three T6SS clusters in *Pseudomonas aeruginosa* suggesting that this RNA regulator imposes a tight and coordinated control [60]. One of the three T6SS of *P*. *aeruginosa*, the H3-T6SS, is similar to T6SS4 of *Y*. *pseudotuberculosis*. It also displays higher levels at 25°C, and is under RsmA/CsrA repression in the opposite to the T3SS of *P*. *aeruginosa* [61, 62], suggesting a more general control mechanism.

The regulation of the T6SS4 cluster is quite complex and underlies highly sophisticated feedback control mechanisms to allow use of T6SS only under specific circumstances (**Fig 10**). The fact that expression of *rovC* is also under dual and antagonistic control by CsrA (positively on the post-transcriptional level and negatively on the transcriptional level) adds another level of complexity, which is most likely important to fine-tune the expression of *rovC* and T6SS4. Based on our results, we propose a dynamic model in which CsrA regulate the expression of *rovC* and T6SS4 in cooperation with RovM in response to the availability of nutrients and ions in the environment. The RovM binding region was mapped from -289 to -319 upstream of the initiation codon of the first T6SS4 ORF [26], closely adjacent to the RovC binding site. As presence of RovC is required for RovM-mediated activation, it is likely that RovC-promoted DNA binding and bending is a prerequisite for RovM binding to the T6SS4 promoter region, leading to a superimposed activation process. In this context, it is surprising, that neither RovM and nor RovC, which binds in close vicinity to the -35 region of the *rovC* promoter (**Figs 3A and 10**), influence *rovC* expression (**S5D and S6 Figs**).

The identified control circuit and T6SS4 regulatory network is further modulated by other environmental cues and regulatory factors. It has previously been shown that temperature and population density/quorum sensing influence T6SS4 expression. Expression of the T6SS4 operon in *Y*. *pseudotuberculosis* and the homologous cluster in *Y*. *pestis* (e.g. KIM y3658-y3677, CO92 YPO0499-YPO0516) is significantly increased at 25°C during stationary phase. This is reflected by the abundance of the secreted protein Hcp in the supernatant at 25°C, whereas at 37°C, Hcp is not detectable [17, 18, 63, 64, 65]. In particular the acylated homoserine lactone (AHL) synthetase YpsI, but also weakly YtbI, contribute to the activation of T6SS4 under these growth conditions [17]. Moreover, the osmolarity and bile salt-responsive response regulator OmpR was previously shown to bind directly to the T6SS4/*rovC* region and activate T6SS4 transcription [19, 20]. In this context, three independent OmpR binding sites were identified of which operator site O_1_ and O_3_ induce the expression of the operon. The O_1_ site is located at position -310 to -290 relative to the transcriptional start site of T6SS4 within the *rovC* coding sequence and O_3_ is at position -45 to -24 and overlaps with the RovC site. Both sites are important for OmpR-mediated T6SS4 induction, whereas unexpectedly, the O_2_ site located at -132 to -112 had a negative influence on T6SS4 expression [19]. This repressive effect can now be explained by an inhibition of the transcription of the RovC activator, as O_2_ is directly positioned between the *rovC* promoter and the start codon (**Fig 10**).

Recent investigation of T6SS4 expression further demonstrated that also the Zn^2+^-responsive regulator ZntR and OxyR, a global oxidative stress regulator activate the T6SS4 gene cluster [21, 22]. As the T6SS4 system was found to function as transporter of the Zn^2+^-chelating protein YezP (YPK_3549) encoded by the operon, ZntR and OxyR-promoted activation helps the bacteria to maintain intracellular zinc homeostasis to fight against nutritional immunity and control the concentration of reactive oxygen species upon oxidative stress [21, 22]. Both activators induce T6SS4 expression directly whereby the ZntR protein was shown to interact with a sequence overlapping the *rovC* coding sequence [22] (**Fig 10**).

The plethora of cross-regulating nutrient-, ion-, and environmental stress-responsive transcription highlight the complexity of the regulatory network regulating *rovC* and the T6SS4 operon and illustrate the importance to fine-tune their expression based on various environmental cues. Given that yersiniae are found in a wide variety of environmental niches and insect vectors (e.g. fleas, flies) and RovC homologues are found in several insect and plant pathogens (**S4A Fig**), it is likely that the *Yersinia* T6SS4 system, besides its described role during infection of mammals [21], is important in the species' native environment. In fact, a recent study comparing gene expression patterns of *Y*. *pestis* and *Y*. *pseudotuberculosis* during infection in the flea digestive tract revealed that the expression of the *Y*. *pseudotuberculosis* T6SS4 system, including the *rovC* homologous gene is strongly upregulated during flea infection compared to *in vitro* growth [66]. It is still unclear how the different regulators and regulatory circuits are integrated and coordinated to produce active T6SS4 when needed. We assume that a concerted action of multiple activators or higher concentrations of an individual activator are required to fully alleviate transcriptional repression of the T6SS4 operon. Based on the environmental cues inducing this type VI secretion machinery and the complexity of the regulatory network controlling its expression it currently appears that this secretion device has evolved as a surveillance, resistance and attack mechanism able to persist and fight interbacterial competition in any ecological niche encountered during their lifestyle.

## Experimental procedures

### Cell culture, media and growth conditions

Overnight cultures of *E*. *coli* were routinely grown at 37°C, *Yersinia* strains were grown at 25°C or 37°C in LB (Luria Bertani) broth if not indicated otherwise. If necessary, antibiotics were added at the following concentrations: carbenicillin 100 μg ml^-1^, chloramphenicol 30 μg ml^-1^ and kanamycin 50 μg ml^-1^.

### Strain and plasmid constructions

All DNA manipulations, restriction digestions, ligations and transformations were performed using standard genetic and molecular techniques [67, 68]. Plasmid DNA was isolated using QIAprep Spin Miniprep Kit (Qiagen). DNA-modifying enzymes and restriction enzymes were purchased from Roche or New England Biolabs. The oligonucleotides used for amplification by PCR, sequencing and primer extension were purchased from Metabion and Eurofins. PCRs were done in a 50 μl mix for 30 cycles using Phusion High-Fidelity DNA polymerase (New England Biolabs). Purification of PCR products was routinely performed using the QIAquick PCR Purification Kit (Qiagen) or the NucleoSpin Gel and PCR Clean-up (Macherey-Nagel). All constructed plasmids were sequenced by Seqlab and Eurofins.

Strains and plasmids used in this study are listed in **S4 Table** and primers for plasmid generation are listed in **S5 Table**. The *rovC*^+^ fragment of *Y*. *pseudotuberculosis* was generated by PCR using primers III286 and III287 (pSSE11), digested with *Bam*HI and *Sal*I, and inserted into pACYC184. Primers V993 and V995 were used to amplify the *rovC* gene by PCR, which was cut with *Sal*I and *Hind*III and cloned under the control of the arabinose inducible promoter P_*BAD*_ of pBAD30 (pVK25).

The His_6_-SUMO-TEV fragment was amplified with primer SUMO TEV and SUMO His from pVK14 and cloned into the *Not*I/*Nco*I site of pCOLA Duet generating pPS041. Subsequently, the *rovC* gene was amplified from chromosomal DNA of YPIII using primer RovC-M1-notI_f and RovC_L247_kpnr and cloned into the *Not*I/*Kpn*I site of pPS041 generating pPS042. Amino acid substitutions were introduced by in vitro mutagenesis using primers PS109-PS161 generating pPS084-107.

For the construction of the His_6_-SUMO-TEV*-rovC*^+^ plasmid pVK14, *rovC* was amplified with primer pair VI157/VI158 and cloned into the *Bam*HI/*Xho*I site of pET28a. For the construction of the *rovC*^+^ plasmid pVK46 and all *rovC*^+^ fragments containing amino acid substitutions (pVK47-pVK52), the *rovC* gene fragment was amplified with primer pair VII533/VII534 and cloned into the *Kpn*I and *Sma*I restriction sites of pZA24 under the control of the P_*ara*_ promoter. To generate the amino acid substitutions, a 2-step PCR was performed, using either the forward primer III286 and a reverse primer containing the mutated sequence or a forward primer with the mutated sequence and the reverse primer III287 (**S5 Table**). Subsequently, both PCR fragments were used as template to amplify the *rovC* gene including the mutation using primer pair VII533/VII534. For construction of a translational *rovC’-‘lacZ* reporter fusion (pVK43), the *rovC* gene was amplified by PCR using primer pair VII244/VII395, digested with *Nhe*I and *Eco*RI and cloned into the *Nhe*I/*Eco*RI site of pBAD18*-lacZ*.

The *rovM*^+^ fragment was generated with primer pair 498/455 and was cloned into the *Sal*I/*Xba*I site of pIV2 to generate pAKH64. Plasmid pSSE32 was generated by the insertion of a PCR fragment, amplified with primer pair III779 and III780, into the *Bam*HI site of pTS02. For the construction of plasmids pSSE64 and pAKH189, PCR fragments were generated with primer pairs IV735/IV736 and III779/V819. The fragments were inserted into the *Bam*HI and *Sal*I sites of plasmids pTS02 or pTS03. The sequence and the correct orientation of the fragments were proven by DNA sequencing.

### Construction of the *Y*. *pseudotuberculosis* deletion mutants

The *Y*. *pseudotuberculosis* strains used in this study are derived from wild-type strain YPIII. All deletion mutants were generated via homologous recombination. First, the kanamycin gene (for deletion of *rovC* in YPIII generating YP148) or the chloramphenicol resistance gene (for deletion of *rovC* in strain YP53 (YP318) and YP72 (YP338) was amplified with primer pairs I661/I662 (for primers see **S5 Table**). Next, the *Yersinia* genomic DNA was used as a template to amplify 500-bp regions flanking the target gene. The upstream fragment was amplified with a primer pair (III920/III921) of which the reverse primer contained additional 20 nt at the 5´-end which were homologous to the start of the kanamycin or chloramphenicol resistance gene. The downstream fragment was amplified with a primer pair (III922/III845) of which the forward primer contained additional 20 nt at the 5´-end which were homologous to the end of the kanamycin or chloramphenicol resistance gene. Subsequently, a PCR reaction was performed with the forward primer of the upstream fragment and the reverse primer of the downstream fragment using the upstream and downstream PCR products of the target gene and the *kanamycin* (or *chloramphenicol*) PCR fragments as templates. The PCR fragments were digested with *Sac*I and were ligated into the *Sac*I site of the suicide plasmid pAKH3 generating plasmid pSSE35 or pVK10. Next, the plasmids were transformed into S17-1λpir and pSSE35 was transferred into *Y*. *pseudotuberculosis* YPIII whereas pVK10 was transferred into YP53 and YP72 via conjugation. Chromosomal integration of the plasmid was selected by plating on LB supplemented with kanamycin or chloramphenicol. Mutants were subsequently grown on LB agar plates containing 10% sucrose. The kanamycin resistance gene of YP148 to generate YP154 was removed as described [69].

Generation of the YPIII strain harboring a chromosomally integrated N-terminal 3x FLAG-tagged *hcp* gene (YP360) was performed using homologous recombination. First, YPIII genomic DNA was used as a template to amplify 500-bp regions flanking the target gene. The upstream fragment was amplified with a primer pair (VI699/VI723) of which the reverse primer contained additional 20 nt at the 5´-end, which were homologous to the 5’-end of a G-block encoding a 3xFLAG-tag. The downstream fragment was amplified with a primer pair (VI724/VI700) of which the forward primer contained additional 20 nt at the 5´-end homologous to the end of the upstream fragment. Then, a PCR reaction was performed with the forward primer of the upstream fragment and the reverse primer of the downstream fragment using the upstream and downstream PCR products of the target gene and the G-block encoding the 3xFLAG-tag as template. The PCR fragments were digested with *Sac*I and were ligated into the *Sac*I site of the suicide-plasmid pAKH3 (pVK30). Next, the plasmid was transformed into S17-1λpir and was transferred into *Y*. *pseudotuberculosis* YPIII via conjugation. Chromosomal integration of the plasmid was proven by PCR using primer homologous to regions upstream of the upstream fragment and downstream of the downstream fragment. Selected PCR fragments were proven by DNA sequencing. Mutants were subsequently grown on LB agar plates containing 10% sucrose. PCR and DNA sequencing were used to prove selected colonies.

A chromosomal replacement of *clpV* by *clpV-gfp* was performed by homologous recombination using the suicide vector pAKH3. For this, fragments for a splice-overlap-extension (SOE)-PCR were generated using primers VIII886-VIII891 and either YPIII genomic DNA or eGFP DNA was used as a template. *Sph*I/*Xma*I-digested pAKH3 and PCR fragments were added to a Gibson assembly reaction (NEB, #E2611S) following manufacturers instructions. The construct was transformed into S17-1λpir and tested for correct inserts by colony PCR and Sanger sequencing using primers III981 and III982. The plasmid was transferred into YPIII and YP53 (Δ*csrA*) via conjugation. Chromosomal integration of the plasmid was confirmed by PCR. Mutants were subsequently grown on LB agar plates containing 6% sucrose. Correct integration of the replaced gene was confirmed by PCR and Sanger sequencing.

### RNA isolation and northern detection

Bacterial cultures were grown under the desired conditions. The bacteria were pelleted by centrifugation for 1 min at 12000 rpm. The pellets were resuspended in 0.2 volume parts of stop solution (5% water-saturated phenol, 95% ethanol) and immediately snap-frozen in liquid nitrogen. After thawing on ice, bacteria were pelleted for 1 min at 12000 rpm at 4°C. The pellet was resuspended in lysozyme solution (50 mg lysozyme/ml TE-buffer) and incubated for at least 5 min at RT. Total RNA of lysed bacteria was isolated with the 'SV Total RNA Isolation System' (Promega, USA) according to the manufacturer’s instructions. RNA concentration and quality were determined by measurement of A_260_ and A_280_.

Total cellular RNA (5–10 μg) was separated on MOPS agarose gels (1.2%), transferred in 10 x SSC buffer (1.5 M NaCl, 0.15 M sodium citrate pH 7.0) for 1.5 h at a pressure of 5 cm Hg to a positively charged nylon membrane by vacuum blotting and UV cross-linked. Prehybridization, hybridization to DIG-labeled riboprobes and membrane washing were conducted using the DIG luminescent Detection kit (Roche) according to the manufacturer’s instructions. The *rovC* transcripts were detected with a DIG-labeled PCR fragment (DIG-PCR nucleotide mix, Roche) with primer pair III286/IV91 (**S5 Table**).

### Primer extension analysis

*Y*. *pseudotuberculosis* YPIII and YP53 were grown to early stationary phase (OD_600_ of 2.0) at 25°C. Total RNA of the samples was prepared using the SV Total RNA Isolation System (Promega). 1–3 μM of a Digoxigenin-labeled primer specific for the *rovC* gene (primer VI17*, **S5 Table**) were annealed with 20 μg RNA in 20 μl of reverse transcriptase buffer by heating to 80°C for 10 min and cooling to 42°C in 1 h. For primer extension, 20 μl annealing reaction mixture was supplemented with 8 mM dNTPs and 1 U SuperScript III Reverse Transcriptase (Thermo Scientific) and incubated for 1 h at 42°C. Sequence ladders were generated with primer VI17* and plasmid pSSE11 as template using the Thermo Sequenase Cycle Sequencing kit (USB) according to the manufacturer´s instructions. Products were analyzed on 6% polyacrylamide gels containing 7 M urea.

### RNA stability assay

*Y*. *pseudotuberculosis* strains YP148 and YP318 harboring plasmid pVK25 were grown at 25°C to exponential phase. Expression of the pBAD promoter was induced by addition of 0.1% arabinose. After 2 h, rifampicin was added in a final concentration of 1 mg/ml to block transcription, and samples were taken in intervals of 0, 2.5, 5, 7.5, and 10 min after addition of rifampicin. To finally analyze the decay rate of the RNA transcripts, northern blots were performed with a *rovC* specific probe, and the detected relative amount of RNA in each sample was determined using ImageJ.

### Quantitative real-time PCR (RT-PCR) analysis

qRT-PCR was performed in triplicates with RNA isolated from at least biological duplicates and was carried out in a Rotor-Gene Q real-time PCR cycler (Qiagen, Germany). Total RNA was isolated using the SV total RNA isolation kit (Promega). qRT-PCR analysis was performed with the 'SensiFast SYBR no-ROX One-step' kit (Bioline) applying the three-step cycling protocol according to the manufacturer. Gene specific-primers used for qRT-PCR amplification are listed in **S5 Table**. The amount of PCR products was quantified by measuring fluorescence of SYBR Green dye. The *sopB* gene was used as reference gene, since it exhibited identical expression levels in the wildtype strain and the tested mutant strains. For each primer-pair a non-template control containing RNase-free water instead of an RNA template was used. Standard curves were detected during every run for each tested gene and established by comparing transcript levels in serial dilutions of total RNA from a control sample. The relative expression of each gene was calculated as described [70].

### Expression and purification of the *Y*. *pseudotuberculosis* RovC wildtype and mutant protein

Plasmid pPS042 encoding the *rovC* wildtype gene as well as its derivatives encoding *rovC* mutations were transformed into the *E*. *coli* strain Rosetta2 (DE3) (Invitrogen) and grown at 37°C in double yeast tryptone (DYT) broth medium supplemented with 30 μg/ml kanamycin and 34 μg/ml chloramphenicol to an OD_600_ of 0.5–0.6. Expression of *rovC* was induced by adding Isopropyl-beta-d-1-thiogalactopyranoside (IPTG) (Carbosynth-EI05931) to a final concentration of 0.12 mM and the bacteria were cultured for another 20 h at 16°C, 130 rpm. The cells were harvested by centrifugation at ∼4690 × g (5000 rpm, Fiberlite F9-4 × 1000y) for 15 min at 4°C and lysed by homogenization at 16000 psi in lysis buffer (100 mM TRIS-HCl pH 8.0, 500 mM NaCl, 5 mM beta-mercaptoethanol (BME), 0.1% Triton X-100, 5 mM MgCl_2,_ 1 mM PMSF, DNase (40 μl at 1 mg/ml per liter of culture)). The lysate was cleared by centrifugation at ∼34,780 × g (15500 rpm, SA-600) at 4°C for 50 mins. RovC was extracted by Ni^2+^-affinity chromatography in batch using Ni^2+^-sepharose resin (GE Healthcare) (pre-equilibrated with loading buffer: 100 mM TRIS-HCl pH 8.0, 500 mM NaCl and 5 mM BME) for 1 h at 4°C with gentle shaking. After washing steps with stepwise increasing the imidazole concentration using wash buffer (100 mM TRIS-HCl pH 8.0, 500 mM NaCl, 5–20 mM imidazole pH 8.0, 5 mM BME), the RovC protein was eluted in elution buffer (100 mM TRIS-HCl pH 8.0, 500 mM NaCl, 100 mM Imidazole pH 8.0, 5 mM BME). Elution fractions were pooled and concentrated using a 10 kDa molecular weight cut-off vivaspin concentrator (GE Healthcare). Concentrated samples were dialyzed in a 3.5 kDa molecular weight dialysis tube in TEV cleavage buffer (100 mM TRIS-HCl pH 8.0, 500 mM NaCl, 5 mM BME, 5% glycerol) and the N-terminal His_6_-Sumo tag was removed overnight by TEV cleavage (ratio 1 mg TEV protease to 30 mg RovC) at 4°C for 15 h. The fusion tag, TEV protease and uncleaved RovC were removed by reverse Ni^2+^-affinity chromatography. The flow through fraction was concentrated and RovC was purified by size-exclusion chromatography (SEC) on a HiLoad 16/60 Superdex 200 column (GE Healthcare), which was pre-equilibrated with SEC buffer containing 50 mM HEPES pH 7.4, 500 mM NaCl, 5% glycerol and 5 mM DTT. The purified RovC was concentrated to 20 mg/ml and stored at -80°C.

### Expression and purification of the *Y*. *pseudotuberculosis* SeMet-labeled RovC protein

For expression of SeMet-labeled RovC, transformed cells were first grown in 2 x 500 ml of DYT medium (supplemented with 30 μg/ml kanamycin and 34 μg/ml chloramphenicol) at 37°C, 130 rpm overnight. 500 ml of fresh DYT medium (supplemented with 30 μg/ml kanamycin and 34 μg/ml chloramphenicol) was added to each of the overnight cultures and cells were grown for another hour at 130 rpm, 37°C. Cells were harvested by centrifugation at 6000 rpm, 4°C for 5 mins and washed twice with minimal medium (per liter: 1 g NH_4_Cl, 3 g KH_2_PO_4_, 4 g Na_2_HPO_4_, 22 g glucose, 0.61 g MgSO_4_, 11.2 mg thiamine-HCl, 10.4 mg Fe_2_(SO_4_)_3_•7 H2O) by centrifugation at 6000 rpm, 5 min at 4°C. The pellet was resuspended in minimal medium and used to inoculate 4 L of Se-Met Medium (minimal medium + 50 mg/l of selenomethionine (Acros Organics)) to an OD_600_ of 1.0. Induction of expression, cell harvest and protein purification were performed as described above.

### Crystallization and structure determination of RovC

RovC was crystallized in 0.2 M KCl, 0.01 M MgSO_4_, 0.01 M MES pH 5.6, 10% PEG400 solution. The crystals were grown in sitting drop at 20°C (0.2 μl of 6 mg/ml protein + 0.2 μl of crystallization solution). For cryoprotection, the crystallization solution was supplemented with additional 20% glycerol. The native data set was collected to 2.3 Å. Since Molecular Replacement failed, SeMet-labeled variant of RovC was purified and crystallized for anomalous phasing methods. SeMet-labeled RovC was crystallized in 0.1 M TRIS pH 8.3, 3.7 M sodium formate with 3 mg/ml of protein at 4°C. For cryoprotection, the crystallization solution was supplemented with additional 20% glycerol. Data for the SeMet crystals were collected to 3.0 Å. Phases were calculated using Phenix-Autosol [71]. An initial model was built using Phenix-Autobuilt [71] and then manually rebuilt in WinCoot [72] followed by refinement in Phenix-Refine [73]. PyMol (www.pymol.org) has been used to generate figures and to calculate the electrostatic surface potential. The structural data has been deposited at the PDB with the PDB-code: 6XZ5. Amino acid alignment were generated with GeneDoc [74].

### Size exclusion chromatography and multi angle light scattering

For the molecular mass determination of RovC, an inline SEC (Akta PURE, GE Healthcare) coupled with a MALS detector (miniDAWN TREOS, Wyatt Technology, wavelength: 658.8 nm) and a differential refractometer (RI) (Optilab T-rEX, Wyatt Technology, wavelength: 658.0 nm, dn/dc (mL/g): 0.1850) was used at room temperature. 2.8–3.3 mg/ml of RovC (or RovC mutants) was loaded on Superdex 200 10/300 GL column prequilibrated with 100 mM TRIS pH 8, 500 mM NaCl and 5 mM DTT. Molecular weight of the eluted protein was analyzed by ASTRA 6 software (Wyatt Technology) by recording RI and MALS signals.

### Small angle X-ray scattering

Synchrotron SAXS data (*I*(*s*) vs *s*, where *s* = 4πsin*θ*/λ, 2*θ* is the scattering angle and λ = 0.124 nm) were measured from a sample of RovC and a corresponding solvent blank (50 mM TRIS, pH 8.0, 500 mM NaCl, 5 mM DTT and 5% v/v glycerol) on the EMBL bioSAXS-P12 beam line at PETRAIII, Hamburg, Germany [75]. An automated sample changer [76], in continuous-flow mode, was used to measure a concentration series of the protein spanning 0.63–5 mg/ml (30 μl sample at 20°C; 1.8 mm pathlength). The sample and buffer measurements were recorded for a total exposure time of 1 s, measured as 20 × 50 ms data frames on a Pilatus 2M area detector. Those frames affected by X-ray radiation damage were identified and discarded from the subsequent automated data reduction steps using the *SASFLOW* pipeline (2D-to-1D radial averaging and buffer subtraction) [77].

Further processing and evaluation of the SAXS data were performed using the *ATSAS* 2.8 software package [78]. The SAXS data reported here were obtained by merging the 5 mg/ml and 2.5 mg/ml data sets in *PRIMUS* [79]. The low-angle data measured from the 2.5 mg/ml sample (0.073 < *s* < 0.63 nm^-1^) were scaled and merged with the high-angle data from the 5 mg/ml profile (0.23 < *s* < 2.6 nm^-1^). The extrapolated forward scattering intensity at zero angle, *I*(0), and the radius of gyration, *R*_*g*_, were determined from the Guinier approximation ([80]; ln*I*(*s*) vs *s*^2^, for *sR*_*g*_ < 1.3) while the probable distribution of real-space distances (*p*(*r*) profile) was calculated using *GNOM* [81] that also provided estimates of the maximum particle dimension, *D*_*max*_. The working *s*_min_–*s*_max_ data range and the number of Shannon channels i.e., the information content of the data taking into account the variance in the scattering intensities and the level of over sampling, were evaluated using *SHANUM* [82]. The data were also classified into shape categories (*DATCLASS* [78]) and concentration-independent molecular weights were estimated directly from the scattering profiles using several scattering-invariant methods (i. SAXSMoW [83], ii. volume of correlation Vc [84], and iii. Porod volume; [85]). A Bayesian analysis, *DATBAYES* [86], was employed to calculate the consensus MW and MW credibility interval using these different approaches. All structural parameters are reported in **S3 Table**. The SAXS data measured for each individual concentration, with an accompanying report, as well as additional data measured from a separate sample at the ESRF-BM29 beam line [87] are made available in the Small Angle Scattering Biological Data Bank (SASBDB, [37]) entry SASDHP5.

### *Ab initio* and rigid-body modelling of RovC

The low-resolution structure of RovC was obtained using the *ab initio* modelling programs *GASBOR* [88] and *DAMMIN*. For *DAMMIN*, ten individual bead models were generated that fit the merged SAXS data (assessed using the reduced *χ*^2^ test and the Correlation Map, or CorMap, *P*-value [77]). The individual models were spatially aligned using *SUPCOMB* ([88]) and subsequently averaged and bead occupancy-corrected with *DAMAVER* [89] to obtain a consensus-low resolution structure (3.7 nm; as assessed using *SASRES* [81] with an average normalized spatial discrepancy, NSD, across the model cohort of 0.6 [81]). GASBOR was also run ten times to produce dummy residue models that fit the merged SAXS data set (model cohort NSD = 1.1). In addition, *CORAL* rigid-body modelling [85] was performed taking the atomistic structure of the RovC monomer obtained from X-ray crystallography and incorporating dummy atom amino-acid linkers to represent those missing regions of unresolved mass in the structure (ca. 20%). Both the positioning of the RovC protomers in the hexameric assembly and the position of the ‘missing mass’ were refined against the SAXS data in P32 symmetry. The final fits to the data of the RovC *CORAL* atomistic models were calculated using *CRYSOL* [90]. Spatial alignments between the *CORAL* and the *GASBOR* or *DAMMIN* models were calculated using *SUPCOMB* [91]. All *DAMMIN*, *GASBOR* and *CORAL* models and their corresponding fits to the merged SAXS data are available in the SASBDB entry SASDHP5. The quality-of-fit estimates of the modeled scattering to the experimental data are also reported in **S3 Table**.

### Expression and purification of the *Y*. *pseudotuberculosis* CsrA protein

For CsrA overexpression, *E*. *coli* strain BL21λDE3 was transformed with pAKH172. 100 ml of LB growth medium containing kanamycin, were inoculated 1:100 from an overnight culture and incubated for 3 h at 37°C. Protein expression was induced by adding 0.5 mM IPTG for 2 h at 37°C. CsrA-His_6_ purification was performed as described previously [27].

### Gel mobility shift assays with DNA

The DNA fragments of the T6SS4 promoter region with varying length (-384 to +11, -317 to +11, and -251 to +11 relative to the T6SS4 translational start site) were amplified by PCR using the Phusion High-Fidelity polymerase (New England Biolabs). PCR fragments (with a final concentration of 130 fmol) were incubated with increasing amounts of purified RovC protein in DNA binding buffer (10 mM HEPES pH7.4, 40 mM NaCl, 5 mM DTT, 5% glycerol, 1 mM MgCl_2_, 0.1 mg/ml BSA) for 30 min at RT. The samples were mixed with 6 x DNA-loading dye (Thermo Scientific) and loaded onto 4% polyacrylamide gels. DNA-protein complexes were stained with ethidium bromide and visualized under UV-light. *CsiD* DNA from *E*. *coli* was used as negative control (-80 to +156 relative to translational start site of the *csiD* gene). The PCR fragments of the T6SS promoter region were synthesized using the following primer pairs: VI648/IV736, VI649/IV736, VI650/IV736, VI652/IV736 and primer pair 131/132 was used for the amplification of the *csiD* fragment.

### Gel mobility shift assays with RNA

The genomic region of interest was amplified by PCR using a forward primer containing a T7 promoter sequence. The following primers were used: primers V773/V777 for *rovC* (+1 to +77 relative to the transcriptional start site), primers V773/VI209 for *rovC* (+1 to +71 relative to the transcriptional start site), primers V775/V777 for *rovC* (+ 19 to +77 relative to the transcriptional start site) and primers V700/I515 for the *hns* control fragment. The generated PCR products were *in vitro*-transcribed according to the TranscriptAid T7 High Yield Transcription Kit (Fermentas). The RNA transcripts were extracted with phenol:chloroform, precipitated with ethanol and stored in RNase free H_2_O. The RNA samples were incubated for 30 min at 800 rpm with calf intestinal alkaline phosphatase (CIP, New England Biolabs) for 5’ dephosphorylation and were labeled with radioactive γ-P^32^-adenosine 5’-triphosphate (ATP) (SRP-301; Hartmann Analytik, Braunschweig). The RNA concentration was adjusted to a final concentration of 2 nM per reaction in band shift buffer (20 mM Na_2_HPO_4_, 20 mM NaH_2_PO_4_, 10 mM KCl, 2 mM DTT, 5% glycerol) and the RNA was denatured at 70°C for 10 min and cooled down on ice for 5 min. The binding reaction was incubated for 20 min on ice and then separated on 4% TBE gels. The samples were exposed to a phosphor screen overnight, and the radioactive RNA fragments were detected using the Typhoon FLA 9000 (GE Healthcare).

### DNase I footprinting assay

For DNase I footprinting, the digoxigenin (DIG)-labeled primer VI826* (DIG-labeled) and IV735 (revers) was used to amplify a fragment corresponding to position +63 to -306 with respect to the transcriptional start site of the T6SS4. The PCR fragment was purified, incubated with the purified RovC protein in band shift buffer (10 mM HEPES pH 7.4, 40 mM NaCl, 5 mM DTT, 5% glycerol, 1 mM MgCl_2_, 0.1 mg/ml BSA). The PCR products were digested with an appropriate dilution of DNase I, and the resulting products were separated and visualized as described [92]. The protected bands were identified by comparison with a sequence ladder, generated with the same DIG-labeled primer used for the amplification of the fragment by PCR. The plasmid pSSE64 was used as template.

### *In vitro* transcription-translation assay

First, the desired genomic region encoding the protein of interest (*rovC*) was amplified by PCR. The forward primer V773 harbors the T7 promoter sequence and the first part of the *rovC* gene and the reverse primer VII836 harbors a terminator sequence and the last nucleotides of the *rovC* coding sequence. The PCR fragment was purified by phenol:chloroform extraction. The *in vitro* transcription-translation was performed using the Pure Express *In Vitro* Protein Synthesis Kit following the manufacturer’s instructions (New England Biolabs). The samples were incubated with 100 nM purified CsrA protein for 2 h at 37°C. Subsequently, the samples were separated in a 15% SDS-Gel and visualized by Coomassie staining and western blotting.

### Gel electrophoresis, preparation of cell extracts and western blotting

For immunological detection of the RovC protein, *Y*. *pseudotuberculosis* cultures were grown at 25°C to stationary phase (OD_600_ 2–3). Cell extracts of equal amounts of bacteria were prepared and separated on 15% SDS polyacrylamide gels [68]. Subsequently, the samples were transferred onto a PVDF membrane via electro-blotting and probed with the respective antibody as described [24]. For visualization of RovC, a generated polyclonal peptide antibody was used (Davids Biotechnology). FLAG-tagged Hcp protein was detected via a monoclonal FLAG antibody (#F1804, Sigma-Aldrich, 1:1000 dilution) and GAPDH was detected with a monoclonal antibody directed against bacterial GAPDH (#MA5-15738, Invitrogen, 1:2000).

### Protein secretion assay

Bacteria were grown overnight at 25°C in LB medium supplemented with 0.05 mM CaCl_2_ and 0.1% arabinose in case of strains harboring pBAD30 or its *rovC*^+^ derivative pVK25 (pP_BAD_::*rovC*^+^), and grown overnight at 25°C. For the preparation of whole cell lysates, 1 ml of the bacterial culture was pelleted and resupended in 1x SDS sample buffer to OD_600_ = 10. For the supernatants, 2 ml of the overnight culture were pelleted, washed and resuspended in fresh LB and incubated at 25°C for 30 min. The samples were centrifuged, 75 μl of the supernatant was added to 25 μl of 4x SDS sample buffer. All samples were heated for 10 min at 95°C, separated on 15% SDS polyacrylamide gels and further analyzed by western blotting (see above).

### Fluorescent microscopy

Bacteria were grown overnight at 25°C in LB medium, diluted 1/50 in fresh LB medium and grown at 25°C to OD_600_ = 2–3. Bacteria were pelleted and resuspended to OD_600_ = 10 and incubated at 25°C for 3 h. 1 μl culture was placed on a 1% agar pad in 0.5 x PBS and imaged on the Keyence microscope BZ-9000 with the software Bz-II viewer. Images were analyzed using the software BZ-II-Analyzer (Keyence).

### β-galactosidase assays

*Y*. *pseudotuberculosis* strains, carrying *lacZ* reporter fusions of interest were grown overnight for 16 h at 25°C. The β-galactosidase activity of the *lacZ* fusion constructs were measured in permeabilized cells as described previously [67]. The activities were calculated as follows: β-galactosidase activity OD_415_ · 6,75 · OD_600_^-1^·Δt (min)^-1^ · Vol (ml)^-1^.

## Supporting information

S1 Table Data collection and refinement statistics. (PDF)

S2 Table Solubility and oligomerization state of RovC variants. (PDF)

S3 Table SAXS data reporting Table for RovC in solution. (PDF)

S4 Table Bacterial strains and plasmids. (PDF)

S5 Table Oligonucleotides for DNA amplification. (PDF)

S1 Fig Purification and crystallization of RovC. **(A)** Coomassie stained gel of eluted fractions of the purified RovC protein. (**B**) Optimized crystals (right) and SDS-PAGE analysis of crystals (S = protein in solution, C = protein in crystals, M = marker). (**C**) Light scattering chromatogram of RovC. Normalized SEC-MALS profile of native RovC (in red). The dotted lines depict the theoretical molecular weight of a RovC pentamer, hexamer and heptamer (theoretical molecular weight is given next to the respective oligomerization state). RovC thus forms a hexamer in solution, according to the experimentally determined molecular weight of approx. 172.8 kDa.
(PDF)

S2 Fig SAXS data analysis and modelling. **(A)** Merged SAXS data measured from RovC samples at 2.5 and 5 mg/ml in 50 mM TRIS, pH 8.0, 500 mM NaCl, 5 mM DTT and 5% v/v glycerol. The scattering intensities *I*(*s*) (grey squares) are presented on an arbitrary log-scale (a.u). *Inset*: Guinier plot of the scattering intensities at very low angle (0.47 < *sR*_*g*_ < 1.3) and corresponding linear fit (black line; R^2^ > 0.99). (**B**) The *p*(*r*) profile of RovC calculated from the SAXS data showing the frequency of real-space vector lengths in the protein (reciprocal space fit: *χ*^2^ = 1.08; CorMap *P* = 0.44). (**C**) *Ab initio* models derived from DAMMIN (left; cyan spheres) and GASBOR (middle; cyan spheres) spatially superimposed with the X-ray crystal structure (green ribbons). To the right is a CORAL rigid-body model representation (green ribbons) that fits the SAXS data (shown in Panel A, blue line; *χ*^2^ = 1.1; CorMap *P* = 0.25) that includes regions of mass that otherwise remain unresolved in the X-ray crystal structure. Also refer to SASBDB (www.sasbdb.org) entry SASDHP5.
(TIF)

S3 Fig Oligomerization and DNA-binding interfaces in RovC. (**A**) Crystal structure of RovC with domain boundaries (top) and cartoon representation of RovC (below). The N-terminal domain is shown in orange and the C-terminal domain is shown in pink. (**B**) Structure of the RovC dimer subunit in two orientations. The two protomers are shown in blue and cyan. The RovC dimer is formed through interactions via the N- and C-terminal domain. The labeling of the domains corresponds to the N- and C-terminal domain of the respective RovC molecule/protomer (e. g. N1 and C1 for N-terminal and C-terminal domain of protomer 1). The black-bordered box depicts the position of the mutant A237E/G242E in interface I (i). (**C**) Hexameric ring of RovC. The hexameric ring is formed by three RovC dimers through the interaction via the N-terminal domains of the RovC dimers (between N1 and N6, N2 and N3, N4 and N5). The black-bordered boxes depict the position of the mutants I150P and I150P/Y151P in interface II (ii) (between N1 and N6, N2 and N3, N4 and N5).
(PDF)

S4 Fig RovC sequence alignment. (**A**) Alignment of *Y*. *pseudotuberculosis* (*Y*. *pstb*) RovC related proteins (red: 100% conserved amino acids; orange: 80%; yellow: 60%) (WP_002224092—hypothetical protein from *Y*. *pestis* biovar mediaevalis (*Y*. *pestis*); WP_049614373—hypothetical protein from *Y*. *pekkanenii* (*Y*. *pekka*); WP_156293748—hypothetical protein from *Serratia oryzae* (*S*. *oryzae*); WP_161740800—hypothetical protein from *Serratia fonticola* (*S*. *fonti*); WP_115459941.1—hypothetical protein from *Enterobacillus tribolii* (*E*. *triboli*); WP_049848866—hypothetical protein from *Trabulsiella odontotermitis* (*T*. *odonto)*; WP_038162275.1—hypothetical protein from *Trabulsiella guamensis* (*T*. *guamensis*); WP_162080698—hypothetical protein from *Enterobacterium bacterium* (*E*. *bacter)*, and WP_130835776—hypothetical protein from *Erwinia mediterraneensis* (*E*. *medit)*. Alignment generated with GeneDoc [74].]. Percentage values on the right correspond to the sequence identity to RovC of *Y*. *pseudotuberculosis*. (**B**) Conserved regions on the surface of RovC shown in two orientations. Color scheme as in (**A**). The dark blue region corresponds to the elongated stretch with ill-defined density in the structure, to which a conservation could not be mapped to.
(PDF)

S5 Fig *rovC* and T6SS4 expression is controlled by temperature. (**A**) Expression of a translational *rovC’*-‘*lacZ* reporter fusion (pSSE32) was monitored in the *Y*. *pseudotuberculosis* wild type strain (YPIII). β-galactosidase activity (μmol/mg^.^min) was measured in strains grown in LB medium at 25°C or 37°C for 4 h (exponential) or 16 h (stationary). The data represent the mean ± standard deviation of three independent experiments, carried out in triplicates. Data were analyzed by Student’s t-test. (**B**) *Y*. *pseudotuberculosis* YPIII (wildtype) was grown in LB medium at 25°C or 37°C for 4 h (exponential) or 16 h (stationary), and *rovC* transcript levels were analyzed by northern blotting. Total RNA was prepared, separated on 0.7% MOPS agarose gels, transferred onto a nylon-membrane and probed with a digoxigenin (DIG)-labeled PCR fragment encoding the *rovC* gene. 16S and 23S rRNAs were used as loading controls. The *rovC* mutant strain YP148 served as negative control; exp = exponential, stat = stationary growth. (**C**) Temperature-dependent expression of a translational T6SS4*'-'lacZ* (pSSE64) fusion in *Y*. *pseudotuberculosis* YPIII wild type was monitored and analyzed as described above. The data represent the mean ± standard deviation of three independent experiments, carried out in triplicates. Data were analyzed by Student’s t-test; *** P<0.001. (**D**) Expression of a translational *rovC’*-‘*lacZ* reporter fusion (pSSE32) was monitored in the *Y*. *pseudotuberculosis* wild type strain (YPIII) and the Δ*rovC* mutant strain (YP154). Both strains were transformed with the empty vector pACYC184 (pV) or complemented with the *rovC*^+^ overexpression plasmid pSSE11 (pP_*rovC*_::*rovC*^+^). β-galactosidase activity (μmol/mg^.^min) was measured in strains grown over night for 16 h at 25°C in LB medium. The data represent the mean ± standard deviation of three independent experiments, carried out in triplicates. Data were analyzed by Student’s t-test.
(PDF)

S6 Fig Analysis of RovM-dependent *rovC* expression. Expression of a translational *rovC’-’lacZ* fusion encoded by pSSE32 was monitored in *Y*. *pseudotuberculosis* YPIII (wildtype) and the Δ*rovM* mutant strain (YP72) transformed with the vector pIV2 (pV) or the *rovM*^+^ plasmid (pAKH64). β-galactosidase activity (μmol/mg^.^min) was measured after strains were grown in LB medium at 25°C. Data are means and standard deviations of three independent experiments, each performed at least in triplicates. Data were analyzed by Student's t test.
(PDF)
