# Supplementary material for: RovC - a novel type of hexameric transcriptional activator promoting type VI secretion gene expression
Source: PLoS Pathog. 2020 Sep 23;16(9):e1008552. doi: 10.1371/journal.ppat.1008552 (PMC7535981; doi:10.1371/journal.ppat.1008552)
Supplement: S1 Table — (PDF) [file ppat.1008552.s001.pdf]

Table S1: Data collection and refinement statistics.

DATA COLLECTION	RovC (PDB-code: 6XZ5)	RovC SeMet
X-ray source	DESY PETRAIII P11	DESY PETRAIII P11
Wavelength [Å]	1.033200	0.979287
Resolution [Å]	33.44 - 2.30 (2.38 - 2.30)	19.40 - 3.00 (3.10 - 3.00)
Space group	P321	R32
Cell dimensions		
a, b, c [Å]	97.28, 97.28, 46.04	97.67,97.67,156.03
α, β, γ [°]	90, 90, 120	90, 90, 120
R_{merge} [%]	8.3 (56.6)	17.8 (267.5)
R_{meas} [%]	9.6 (65.7)	18.1 (271.9)
$CC_{1/2}$ [%]	99.6 (69.4)	99.9 (85.8)
‹I/σ(I)›	9.7 (2.3)	21.46 (2.2)
Completeness [%]	99.5 (99.9)	99.7 (99.5)
Unique reflections	11309 (1095)	11112 (1039)
Redundancy	4.0 (4.0)	30.9 (31.0)
**REFINEMENT**		
Resolution [Å]	33.44 - 2.30	
R_{work} [%]	22.87 (28.43)	
R_{free} [%]	27.36 (30.94)	
No. of non-hydrogen atoms	1732	
Avg. B factor [$Å^2$]	39.16	
R.m.s. deviations		
Bond length [Å]	0.002	
Bond angles [°]	0.45	
Rotamer outliers [%]	0.00	
Ramachandran [%] (favored/allowed/outliers)	98.33/1.67/0.00	

Statistics for the highest-resolution shell are shown in parentheses.
