# Supplementary material for: RovC - a novel type of hexameric transcriptional activator promoting type VI secretion gene expression
Source: PLoS Pathog. 2020 Sep 23;16(9):e1008552. doi: 10.1371/journal.ppat.1008552 (PMC7535981; doi:10.1371/journal.ppat.1008552)
Supplement: S2 Table — (PDF) [file ppat.1008552.s002.pdf]

Table S2: Solubility and oligomerization state of RovC variants

RovC variant	Solubility	MW MALLS (kDa) / oligomerization state
RovC wt	+	172.8 ± (0.2%)/ hexamer
I150P	+	Mixture/ 3-4 different oligomerization states
I150P/Y151P	--	n.d.
K163E	+	172.8 ± (0.2%)/ hexamer
K175E	+	173.2 ± (0.1%)/ hexamer
R202E	+	171.0 ± (0.2%)/ hexamer
K211E/K215E	+	170.3 ± (0.3%)/ hexamer
S219E/A220E	+	169.1 ± (0.2%)/ hexamer
R225E	+	168.4 ± (0.4%)/ hexamer
A237E	-	n.d.
G242E	-	n.d.
