# Supplementary material for: RovC - a novel type of hexameric transcriptional activator promoting type VI secretion gene expression
Source: PLoS Pathog. 2020 Sep 23;16(9):e1008552. doi: 10.1371/journal.ppat.1008552 (PMC7535981; doi:10.1371/journal.ppat.1008552)
Supplement: S3 Table — (PDF) [file ppat.1008552.s003.pdf]

Table S3: SAXS data reporting Table for RovC in solution

Sample details	
Organism	*Yersinia pseudotuberculosis*
Uniprot ID	A0A0H3B5N9
Concentrations measured	0.63-5 mg/ml
Solvent composition	50 mM TRIS pH= 8, 500 mM NaCl, 5 mM DTT, 5 % v/v glycerol
Temperature (°C)	20
Instrument details	
Instrument	EMBL-P12 BioSAXS
Exposure time/number of frames	1 s/(20 x 50 ms)
X-ray wavelength (nm)	0.124
Method for monitoring radiation damage	CorMap
s-measurement range (nm^{-1})	0.02-5
Information content: Merged 2.5 and 5 mg/ml data	
Method	SHANUM
#Shannon channels	12
Final working *s*-range (nm^{-1})	0.07-3.12
Guinier analysis: Merged 2.5 and 5 mg/ml data	
Primary data analysis software	PRIMUSQT
Guinier $I(0)$ (σ)	9158 (13)
R_g (Guinier, nm) (σ)	4.22 (0.1)
sR_g range/(points used)	0.47-1.3(21-115)
p(r) analysis: Merged 2.5 and 5 mg/ml data	
Method	GNOM5
$I(0)$, POR (σ)	9039 (13)
R_g (POR, nm) (σ)	4.12 (0.1)
D_{max} (nm)	12.4
Quality of fit, CorMap P value/χ^2	0.44/1.08
Porod volume (nm^3)	280
Shape classification	Compact-hollow
MW analysis: Merged 2.5 and 5 mg/ml data	
Calculated MW, from amino acid sequence - hexamer	175 kDa
Concentration-independent MW estimates	
MW (Bayesian estimate, kDa)/probability	186/0.8
MW Credibility Interval (kDa)/probability	162-195/0.98
MW SAXSMow (kDa)	185
MW Vc (kDa)	186
MW Porod (kDa)	175
Ab initio modeling: Merged 2.5 and 5 mg/ml data	
Method	DAMMIN
Symmetry	P32

Quality-of-fit, CorMap *P* value/χ^2	0.01-0.25/1.1
Average NSD (10 model cohort)	0.6
Method	GASBOR
Symmetry	P32
Quality-of-fit, CorMap *P* value/χ^2	0.03-0.68/1.08
Average NSD (10 model cohort)	1.1
Rigid body: Merged 2.5 and 5 mg/ml data	
Refinement Method	CORAL
Symmetry	P32
Fitting method	CRYSOL
#harmonics/points/*s*-max (nm^{-1}) for fitting	30/200/2.6
Constant adjustment	Yes
Quality-of-fit, CorMap *P* value/χ^2	0.25/1.1
Data availability/Accession code(s)	
SASBDB	SASDHP5
