# Supplementary material for: RovC - a novel type of hexameric transcriptional activator promoting type VI secretion gene expression
Source: PLoS Pathog. 2020 Sep 23;16(9):e1008552. doi: 10.1371/journal.ppat.1008552 (PMC7535981; doi:10.1371/journal.ppat.1008552)
Supplement: S4 Table — (PDF) [file ppat.1008552.s004.pdf]

Table S4: Bacterial strains and plasmids

Strains, plasmids	Description	Source and reference
**Bacterial strains** *E. coli* **K12**		
BL21λDE3	F⁻ *ompT gal dcm lon hsdSB*(rB - mB -) λDE3	[1]
CC118λ*pir*	F⁻ Δ(*ara-leu*)7697 Δ(*lacZ*)74 Δ(*phoA*)20 *araD139 galE galK thi rpsE rpoB arfE*^{am} *recA1*, λ*pir*	[2]
S17-1λ*pir*	*recA thi pro hsdR*⁻ M1⁺(RP4⁻-2Tc::Mu-- Km::Tn7), λ*pir*	[3]
DH5α	F-Φ80*lac*ZΔM15 Δ(*lacZYA-arg*F) U169 *recA1endA1hsdR17* (rk⁻, mk⁺) *phoA supE*44λ - *thi*-1 *gyrA*96 *relA1*	Invitrogen
Rosetta2 DE3	F- *ompT hsdS$_B$* (r_B^- m_B^-) *gal dcm* (DE3) pRARE2 (Cam^R)	Invitrogen
Y. pseudotuberculosis		
YPIII	pIB, wildtype	[4]
YP53	YPIII, Δ*csrA*, Kan^R	[5]
YP72	YPIII, Δ*rovM*	[6]
YP89	YPIII, Δ*crp*	[6]
YP148	YPIII, Δ*rovC*, Kan^R	This study
YP154	YPIII, Δ*rovC*, ΔKan^R	This study
YP318	YPIII, Δ*csrA*, Kan^R Δ*rovC*, Cm^R	This study
YP338	YPIII, Δ*rovM*, Δ*rovC*, Cm^R	This study
YP360	YPIII, N-terminal 3xFLAG-YPK_3563	This study
YP412	YPIII, *clpV-gfp*	This study
YP417	YPIII, Δ*csrA*, Kan^R, *clpV-gfp*	This study
YP420	YPIII, Δ*csrA*, Kan^R, N-terminal 3xFLAG-YPK_3563	This study
**Plasmids**		
pACYC184	cloning vector, ori p15A, Tet^R, Cm^R	[7]
pAKH3	pGP704, ori R6K, *sacB*⁺, Cm^R	[6]
pAKH56	pACYC184, p15A, *csrA*⁺, Cm^R	[5]
pAKH63	pGP20, ori pSC101, *rovM'-'lacZ* (41)c, Tet^R	[6]
pAKH64	pIV2, *rovM*⁺, *Y. enterocolitica* (p29807), Kan^R	This study
pAKH85	pACYC184, p15A, Δ*tet*, Cm^R	[8]
pAKH172	pET28a(+), ori 3286 ,*csrA*⁺, Kan^R	[9]
pAKH189	pTS03, ori pSC101, *rovC-lacZ* (-579 to +1)[a], Amp^R	This study
pASS89	pAKH3, YPK_3559 (ClpV)-Ala-Gly-linker-*gfp*	This study
pBAD30	cloning vector, ori p15A, Amp^R	[10]
pBAD18-*lacZ*(481)	translational *lacZ* fusion vector, ori pBR322, Amp^R	[11]
pET28a	T7 overexpression vector, Kan^R	Novagen
pIV2	Cloning vector, ori p29807, Kan^R	[12]
pPS041	modified pCOLADuet, Kan^R, N-His6-SUMO-TEV	This study
pPS042	pCOLADuet (pPS041), RovC_M1-L247, Kan^R, N- His6-SUMO-TEV	This study

pPS084	pCOLADuet (pPS041), RovC_M1-L247 (A237E), Kan^R, N-His6-SUMO-TEV	This study
pPS085	pCOLADuet (pPS041), RovC_M1-L247 (S219E/A220E), Kan^R, N-His6-SUMO-TEV	This study
pPS090	pCOLADuet (pPS041), RovC_M1-L247 (G242E), Kan^R, N-His6-SUMO-TEV	This study
pPS092	pCOLADuet (pPS041), RovC_M1-L247 (I150P/Y151P), Kan^R, N-His6-SUMO-TEV	This study
pPS093	pCOLADuet (pPS041), RovC_M1-L247 (I150P), Kan^R, N-His6-SUMO-TEV	This study
pPS094	pCOLADuet (pPS041), RovC_M1-L247 (K175E), Kan^R, N-His6-SUMO-TEV	This study
pPS102	pCOLADuet (pPS041), RovC_M1-L247 (R225E), Kan^R, N-His6-SUMO-TEV	This study
pPS105	pCOLADuet (pPS041), RovC_M1-L247 (K163E), Kan^R, N-His6-SUMO-TEV	This study
pPS106	pCOLADuet (pPS041), RovC_M1-L247 (R202E), Kan^R, N-His6-SUMO-TEV	This study
pPS107	pCOLADuet (pPS041), RovC_M1-L247 (K211E/K215E), Kan^R, N-His6-SUMO-TEV	This study
pSSE11	pACYC184, p15A, *rovC*$^+$, Cm^R	This study
pSSE35	pAKH3, Δ*rovC*, Amp^R	This study
pSSE32	pTS02, ori pSC101, *rovC*'-'*lacZ* (3)^c, Amp^R	This study
pSSE64	pTS02, ori pSC101, YPK_3566'-'*lacZ* (3)^c, Amp^R	This study
pTS02	pGP20, ori pSC101, *lacZ*, Amp^R	[13]
pTS03	pGP20, ori pSC101, RBS-*lacZ*$^+$, Amp^R	This study
pVK10	pAKH3, Δ*rovC*, Cm^R	This study
pVK14	pET28, *rovC*$^+$, Kan^R, N-His6-SUMO	This study
pVK25	pBAD30, p15A, 5'UTR-*rovC* (-39)^b, Cb^R	This study
pVK30	pAKH3, YPK_3563, 3xFLAG-tag, SAGASA-linker	This study
pVK43	pBAD18-*lacZ,* 5'-UTR-*rovC*, ori ColE1, Amp^R	This study
pVK46	pZA24, ori p15A, *rovC*$^+$, Kan^R	This study
pVK47	pZA24, ori p15A, *rovC*$^+$ K163E, Kan^R	This study
pVK48	pZA24, ori p15A, *rovC*$^+$ K175E, Kan^R	This study
pVK49	pZA24, ori p15A, *rovC*$^+$ K211E/K215E, Kan^R	This study
pVK50	pZA24, ori p15A, *rovC*$^+$ R202E, Kan^R	This study
pVK51	pZA24, ori p15A, *rovC*$^+$ R225E, Kan^R	This study
pVK52	pZA24, ori p15A, *rovC*$^+$ S219E/A220E, Kan^R	This study
pZA24	expression vector, P_{ara}, p15A, Kan^R	[14]

[a] the number indicates the nucleotide relative to the transcriptional start site

[b] the number indicates the nucleotide relative to the translational start site

[c] the number indicates the codon of the corresponding gene fused to the reporter gene

References:

1. Studier FW, Moffatt BA. Use of bacteriophage T7 RNA polymerase to direct selective high-level expression of cloned genes. Journal of molecular biology. 1986;189: 113–30.

2. Manoil C, Beckwith J. A genetic approach to analyzing membrane protein topology. Science (New York, NY). 1986;233: 1403–8.
3. Simon R, Priefer U, Pühler A. A Broad Host Range Mobilization System for In Vivo Genetic Engineering: Transposon Mutagenesis in Gram Negative Bacteria. Bio/Technology. 1983;1: 784–791. doi:10.1038/nbt1183-784
4. Bolin I, Norlander I, Wolf-Watz H, I Bölin LN. Temperature-inducible outer membrane protein of Yersinia pseudotuberculosis and Yersinia enterocolitica is associated with the virulence plasmid. Infect Immun. 1982;37: 506-512.
5. Heroven AK, Bohme K, Rohde M, Dersch P. A Csr-type regulatory system, including small non-coding RNAs, regulates the global virulence regulator RovA of *Yersinia pseudotuberculosis* through RovM. Mol Microbiol. 2008;68: 1179–95. doi:MMI6218 [pii] 10.1111/j.1365-2958.2008.06218.x
6. Heroven AK, Sest M, Pisano F, Scheb-Wetzel M, Steinmann R, Bohme K, et al. Crp induces switching of the CsrB and CsrC RNAs in *Yersinia pseudotuberculosis* and links nutritional status to virulence. Front Cell Infect Microbiol. 2012;2: 158. doi:10.3389/fcimb.2012.00158
7. Chang AC, Cohen SN. Construction and characterization of amplifiable multicopy DNA cloning vehicles derived from the P15A cryptic miniplasmid. J Bacteriol. 1978;134: 1141–1156.
8. Heroven AK, Dersch P. RovM, a novel LysR-type regulator of the virulence activator gene *rovA*, controls cell invasion, virulence and motility of *Yersinia pseudotuberculosis*. Mol Microbiol. 2006;62: 1469–83.
9. Kusmierek M, Hoßmann J, Witte R, Opitz W, Vollmer I, Volk M, et al. A bacterial secreted translocator hijacks riboregulators to control type III secretion in response to host cell contact. PLoS Pathog. 2019;15: e1007813. doi:10.1371/journal.ppat.1007813

10. Guzman LM, Belin D, Carson MJ, Beckwith J. Tight regulation, modulation, and high-level expression by vectors containing the arabinose P_{BAD} promoter. J Bacteriol. 1995;177: 4121–30.

11. Waldminghaus T, Fippinger A, Alfsmann J, Narberhaus F. RNA thermometers are common in alpha- and gamma-proteobacteria. Biol Chem. 2005;386: 1279–86. doi:10.1515/BC.2005.145

12. Strauch E, Voigt I, Broll H, Appel B. Use of a plasmid of a *Yersinia enterocolitica* biogroup 1A strain for the construction of cloning vectors. J Biotechnol. 2000;79: 63–72.

13. Böhme K, Steinmann R, Kortmann J, Seekircher S, Heroven AK, Berger E, et al. Concerted actions of a thermo-labile regulator and a unique intergenic RNA thermosensor control *Yersinia* virulence. PLoS Pathog. 2012;8: e1002518. doi:10.1371/journal.ppat.1002518

14. Lutz R, Bujard H. Independent and tight regulation of transcriptional units in *Escherichia coli* via the LacR/O, the TetR/O and AraC/I1-I2 regulatory elements. Nucleic Acids Res. 1997;25: 1203–10.
