# Supplementary material for: RovC - a novel type of hexameric transcriptional activator promoting type VI secretion gene expression
Source: PLoS Pathog. 2020 Sep 23;16(9):e1008552. doi: 10.1371/journal.ppat.1008552 (PMC7535981; doi:10.1371/journal.ppat.1008552)
Supplement: S5 Table — (PDF) [file ppat.1008552.s005.pdf]

Table S5: Oligonucleotides for DNA amplification

Oligonucleotide	Sequence (5'-3')	Restriction site
131	GTCCTGGCCTGAATCGACAGCG	
132	GGCTACGAAATGAGCATCGC	
455	GCG GCG GTC GAC GGT CTT AAA AAG AGA ACA TTC	*Sal*I
498	GCG GCG TCT AGA GCA GGA TCA CCG ATA CTG	*Xba*I
I515	GCAGTTCATTTGGATCAATAC	
I661	GTGTAGGCTGGAGCTGCTTC	
I662	CATATGAATATCCTCCTTAGTTCC	
III286	CGCGCGGTCGACCATATTCAACGCCGAATAATGC	*Bam*HI
III287	CGCGCGGGATCCCTAGAGGAAGTTCAGGTAGCC	*Bam*HI
III393	CCGACGTAAAGCCGCGATAC	qRT *sopB* control
III394	CCTCGTTCATAAGCACTCGTC	qRT *sopB* control
III779	GCGCGGATCCGGCGAAGCGGTCATCAATA	*Bam*HI
III780	GCGCGGATCCTTACTACTCATGGATATCTCC	*BamH*I
III845	GCGCGAGCTCGGCAGAGTTAATGTAATGTTCC	*Sac*I
III920	GCGCGAGCTCGGCTTGCTCACTGATATG	*Sac*I
III921	GAAGCAGCTCCAGCCTACACATCTATGTCCTCTTATTTTGGC	
III922	ACTAAGGAGGATATTCATATGTGTCCTATCTGACATGC	
IV91	GCGCGAGCTCGCTCCTCTTTGCATTCCAC	
IV735	GCGCGGATCCCAGCTCTGATTGGATTAATTCAG	*Bam*HI
IV736	GCGCGTCGACATGTCACTCATATTATTGTCCATC	*Sal*I
V647	ATGTATTTACGGCGTCTTTACGATC	qRT YPK_3548
V648	TTAGATGCTATCCGGCTGGTGG	qRT YPK_3548
V649	GCTCACCTTACGTGCCAGCGT	qRT YPK_3548
V650	CCGCATTATCGATCCACCCTATG	qRT YPK_3548
V651	CGGCCCAACTGGATGTGCTC	qRT YPK_3559
V652	CATGCAGATGGCGGCTTTGC	qRT YPK_3559
V653	CATCTTCGACATTATTTTTAACTGTC	qRT YPK_3559
V654	GTTCACAATGCAGTTGGTAACTC	qRT YPK_3559
V700	GGGCGCGTAATACGACTCACTATAGCAACAATACCGTGAAATGC	T7 promoter
V773	GTAATACGACTCACTATAGGATGACGTGGATGAATAGCC	T7 promoter
V775	GTAATACGACTCACTATAGCAAAATAAGAGGACATAGAT	T7 promoter
V777	CATTCCCACGCGAAGTCATTAT	
V819	GCGCGTCGACGTGCTAATGGTTATGTAAATTT	*Sal*I
V993	GGCGGGTCGACGATGACGTGGATGAATAGCCAA	*Sal*I
V995	GGAAGGAAGCTTCTAGAGGAAGTTCAGGTAGCC	*Hind*III
VI17*	CATAAATAATTCCCAATCGCTAA	
VI157	GCGTAGGATCCGAGAAAGAAGCTATATAATGACTTC	*Bam*HI
VI158	GCGCTCTCGAGTCACTAGAGGAAGTTCAGGTAGCCG	*Xho*I
VI209	CACGCGAAGTCATTATATAGCTTC	
VI648	CGAAGTCATTATATAGCTTCTTTCTC	
VI649	GTGCTAATGGTTATGTAAATTTAATAAATG	
VI650	CTACTACTGTTTTTTGTCCTAGGTTATTTC	
VI699	GGCAAGGAGCTCGGCCCGTTTACCTTACATTTTTG	*Sac*I
VI700	GGCAAGGAGCTCGATCGTAACCAGCCTTAATCACG	*Sac*I
VI723	CGTCATGGTCTTTGTAGTCCATTTTTTCACTCCTGAATTAATG	
VI724	CGATAAATCTGCTGGCGCGAGTGCAGCAGCTTTAGTCGATTACTTTC	
VI826*	CCTGATTTACATACCTGTTATAGG	DNase footprinting
VI917	GCCTTTATCGGGTCGTGCACCCTG	qRT YPK_3563
VI918	GATAACGCAACTCTTCGTTGTATAG	qRT YPK_3563
VII244	GCCGCGCTAGCGATGACGTGGATGAATAGCCAAAATAAG	*Nhe*I
VII248	CTAATGGAGGGGGTATACCCGATG	qRT YPK_3567
VII249	CTGAACGGTTACTTAATTTTAGTGACC	qRT YPK_3567
VII250	CGCGTGTTCAGAATGATTTTCCCAC	qRT YPK_3568

VII251	CCCGTGCAATATAGGTCTTCTTGGG	qRT YPK_3568
VII395	CGGCGCGAATTCATTTCGCCTCAGGCATTCCC	*Eco*RI
VII533	CGCCGGGTACCCATATTCAACGCCGAATAATGC	*Kpn*I
VII534	GCGGCCCCCGGGCTAGAGGAAGTTCAGGTAGCC	*Sma*I
VII836	GAGTTTGTAGAAACGCAAAAAGG	
VIII886	agcttcttctagaggtaccgcatgACTCGGGATCTTCCTGCTGG	
VIII887	GAAAAGTTCTTCTCCTTTACGtcctcctgcggccgccgcCGCGAACTCAATACTCAGTTC	
VIII888	GAACTGAGTATTGAGTTCGCGgcggcggccgcaggaggaCGTAAAGGAGAAGAACTTTTC	
VIII889	CGTTGTCGACTAAACATCAGTTATTTGTATAGTTCATCCA	
VIII890	TGGATGAACTATACAAATAACTGATGTTTAGTCGACAACG	
VIII891	acaatttgtggaattcccggTAAGGAAATCGAAGTCACGT	
PS109	AAGGCGAATGAACTTATTAATTACGGCTACCTGAACTTCCTC	
PS110	ATTAATAAGTTCATTCGCCTTCTTTATACGATAGCGGATTTTCG	
PS111	GGGGTACCTTAGAGGAAGTTCAGGTATTCG	
PS114	GAATGGGAAGAAGACAGTTGGGTCAGAGCAAAAATCCGC	
PS115	ACTGTCTTCTTCCCATTCATTTTTTACCAGTTCCTTACC	
PS123	CTCTATCCTTATATCCCTTTGAACCTTGAATCTGATGTTTTTG	
PS124	AGGGATATAAGGATAGAGGTTTGAATCATCTTTTAATGCATCTGC	
PS125	CTCTATCCTCCTATCCCTTTGAACCTTGAATCTGATGTTTTTG	
PS126	AGGGATAGGAGGATAGAGGTTTGAATCATCTTTTAATGCATCTGC	
PS130	AATCACGAGATAGAGGTGGAATGCAAAGAGGAGCAATATTTAGG	
PS131	CACCTCTATCTCGTGATTAACAATGCTCTGTAATAACTC	
PS150	TGGGTCGAAGCAAAAATCCGCTATCGTATAAAGAAGGCG	
PS151	TTTTGCTTCGACCCAACTGTCAGCAGACCATTCATTTTTTAC	
PS156	GTTTTTGCTGAAAATATTGAGTTATTACAGAGCATTGTTAATCAC	
PS157	ACTCAATATTTTCAGCAAAAACATCAGATTCAAGGTTCAAAG	
PS158	TCCCACGAAGATATCGCTTCAGAAATTTTTGGTAAGGAAC	
PS159	GATATCTTCGTGGGAGAAGCCTTGTTTTCTGTCATC	
PS160	GGTGAAGAACTGGTAGAAAATGAATGGTCTGCTGACAGTTGGG	
PS161	ATTTTCTACCAGTTCTTCACCAAAAATTTCTGAAGCGATATCCCG GTG	
SUMO TEV	ATTCTTGCGGCCGCCTCCCTGGAAGTAGAGGTTCTCGTCGACACCAATCTGTTCTCTGTG	
SUMO His	CATGCCATGGGCCATCATCATCACCATCATTCGGACTCAGAAGTCAATCAAG	
RovC_M1-notI_f	AAGAATGCGGCCGCATGTCCAACCTACCAGGAGTCAAAC	*Not*I
RovC_L247_kpn_r	GGGGTACCTTAGAGGAAGTTCAGGTAGCCG	*Kpn*I

The corresponding restriction sites and the T7 promoter are underlined.

*Digoxygenin labeled
