# Supplementary figures and images for: RovC - a novel type of hexameric transcriptional activator promoting type VI secretion gene expression
Source: PLoS Pathog. 2020 Sep 23;16(9):e1008552. doi: 10.1371/journal.ppat.1008552 (PMC7535981; doi:10.1371/journal.ppat.1008552)

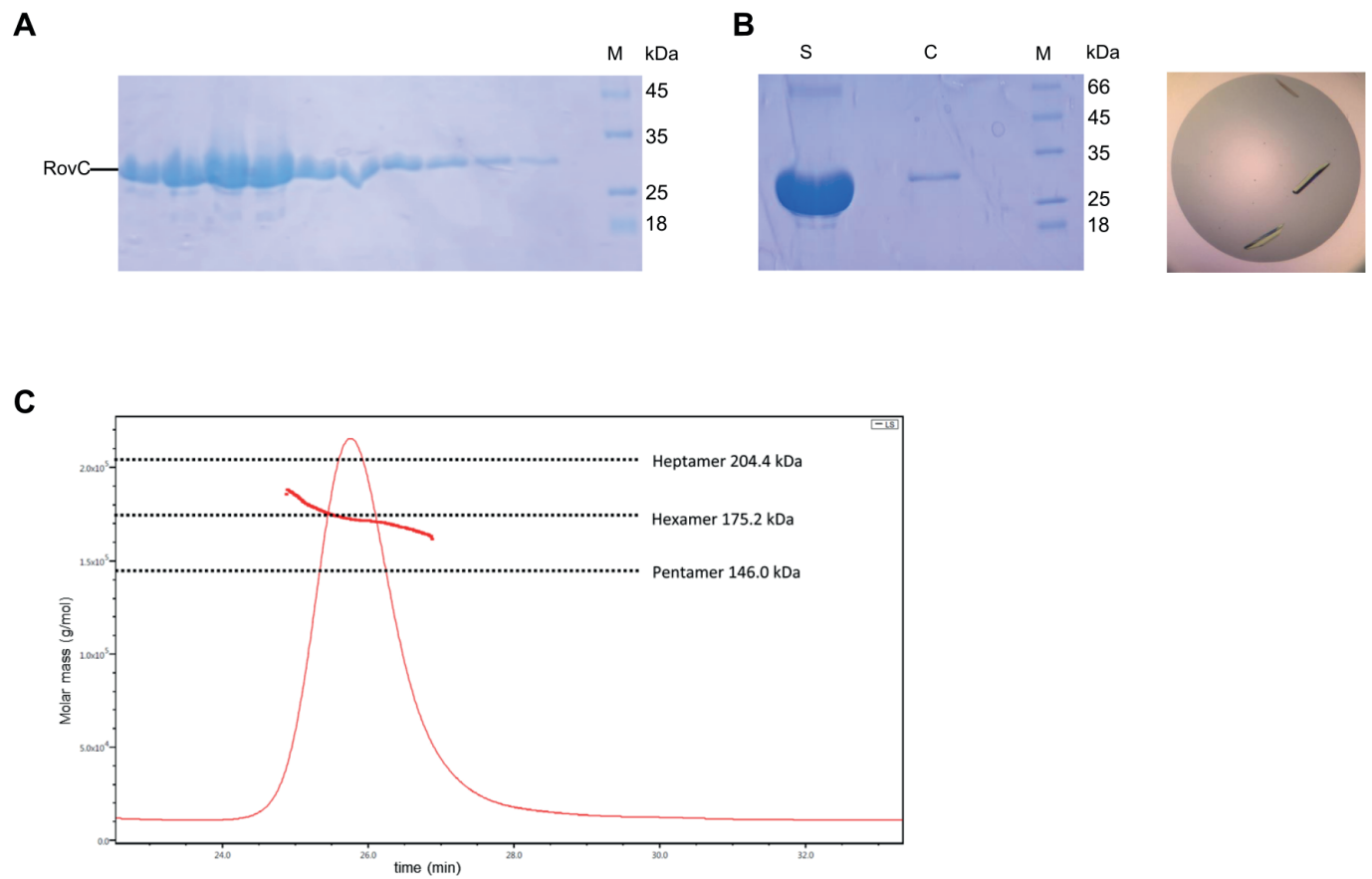

**Figure S1:** Knittel *et al.* 2020

Supplement: S1 Fig — (A) Coomassie stained gel of eluted fractions of the purified RovC protein. (B) Optimized crystals (right) and SDS-PAGE analysis of crystals (S = protein in solution, C = protein in crystals, M = marker). (C) Light scattering chromatogram of RovC. Normalized SEC-MALS profile of native RovC (in red). The dotted lines depict the theoretical molecular weight of a RovC pentamer, hexamer and heptamer (theoretical molecular weight is given next to the respective oligomerization state). RovC thus forms a hexamer in solution, according to the experimentally determined molecular weight of approx. 172.8 kDa. (PDF) [file ppat.1008552.s006.pdf]

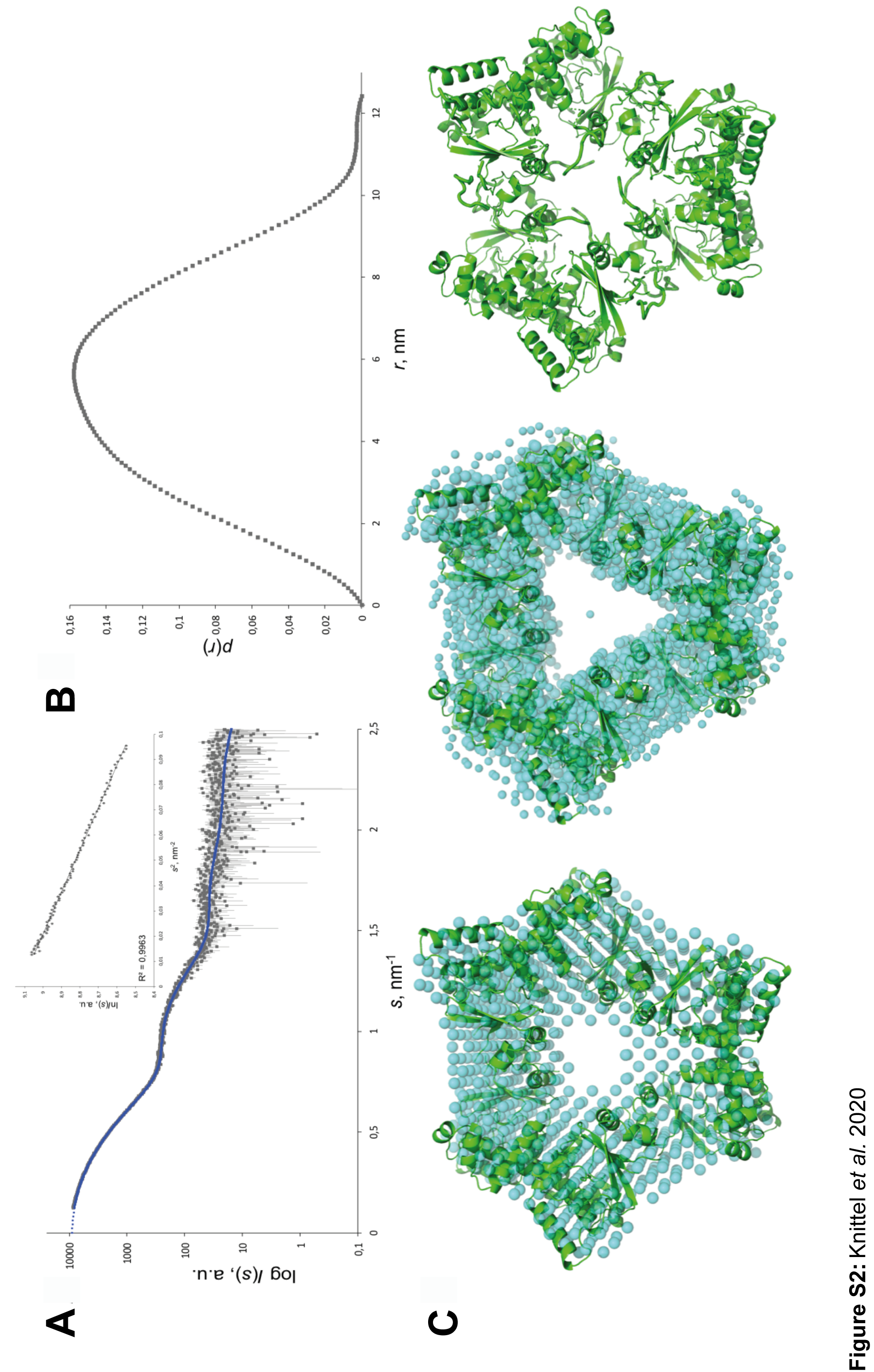

Supplement: S2 Fig — (A) Merged SAXS data measured from RovC samples at 2.5 and 5 mg/ml in 50 mM TRIS, pH 8.0, 500 mM NaCl, 5 mM DTT and 5% v/v glycerol. The scattering intensities I(s) (grey squares) are presented on an arbitrary log-scale (a.u). Inset: Guinier plot of the scattering intensities at very low angle (0.47 < sRg < 1.3) and corresponding linear fit (black line; R2 > 0.99). (B) The p(r) profile of RovC calculated from the SAXS data showing the frequency of real-space vector lengths in the protein (reciprocal space fit: χ2 = 1.08; CorMap P = 0.44). (C) Ab initio models derived from DAMMIN (left; cyan spheres) and GASBOR (middle; cyan spheres) spatially superimposed with the X-ray crystal structure (green ribbons). To the right is a CORAL rigid-body model representation (green ribbons) that fits the SAXS data (shown in Panel A, blue line; χ2 = 1.1; CorMap P = 0.25) that includes regions of mass that otherwise remain unresolved in the X-ray crystal structure. Also refer to SASBDB (www.sasbdb.org) entry SASDHP5. (TIF) [file ppat.1008552.s007.tif]

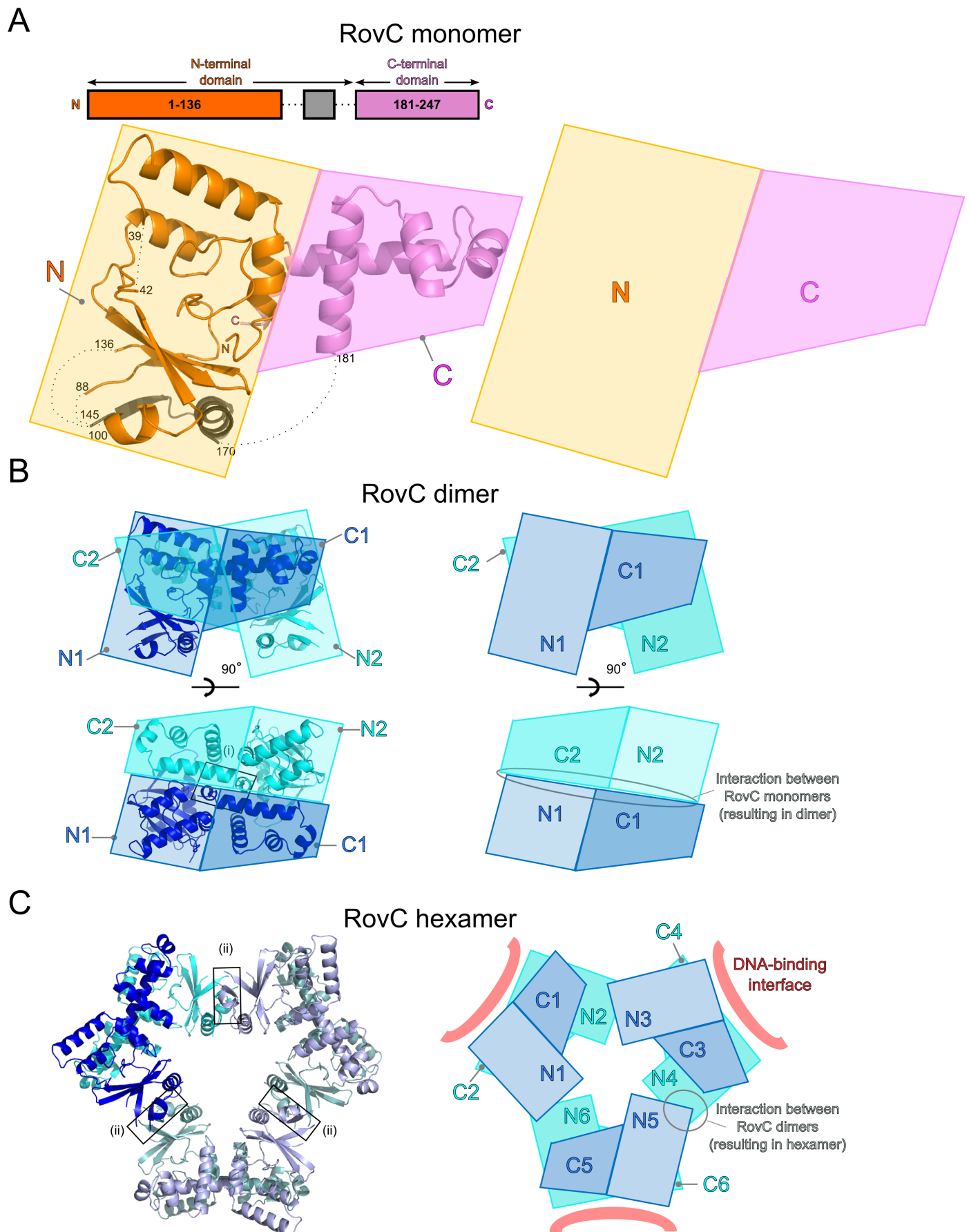

**Figure S3:** Knittel *et al.* 2020

Supplement: S3 Fig — (A) Crystal structure of RovC with domain boundaries (top) and cartoon representation of RovC (below). The N-terminal domain is shown in orange and the C-terminal domain is shown in pink. (B) Structure of the RovC dimer subunit in two orientations. The two protomers are shown in blue and cyan. The RovC dimer is formed through interactions via the N- and C-terminal domain. The labeling of the domains corresponds to the N- and C-terminal domain of the respective RovC molecule/protomer (e. g. N1 and C1 for N-terminal and C-terminal domain of protomer 1). The black-bordered box depicts the position of the mutant A237E/G242E in interface I (i). (C) Hexameric ring of RovC. The hexameric ring is formed by three RovC dimers through the interaction via the N-terminal domains of the RovC dimers (between N1 and N6, N2 and N3, N4 and N5). The black-bordered boxes depict the position of the mutants I150P and I150P/Y151P in interface II (ii) (between N1 and N6, N2 and N3, N4 and N5). (PDF) [file ppat.1008552.s008.pdf]

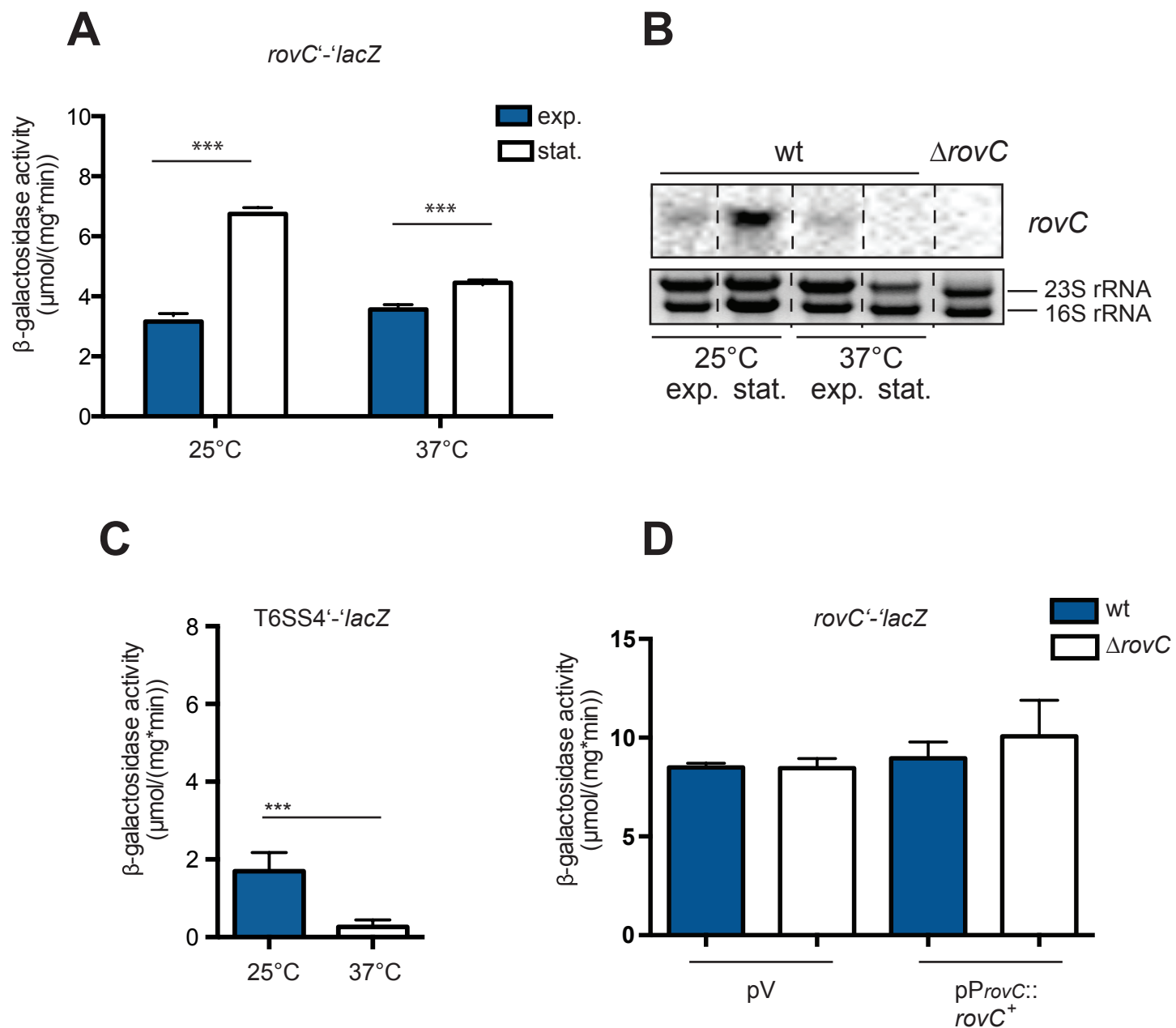

**Figure S5:**Knittel *et al.* 2020

Supplement: S5 Fig — (A) Expression of a translational rovC’-‘lacZ reporter fusion (pSSE32) was monitored in the Y. pseudotuberculosis wild type strain (YPIII). β-galactosidase activity (μmol/mg.min) was measured in strains grown in LB medium at 25°C or 37°C for 4 h (exponential) or 16 h (stationary). The data represent the mean ± standard deviation of three independent experiments, carried out in triplicates. Data were analyzed by Student’s t-test. (B) Y. pseudotuberculosis YPIII (wildtype) was grown in LB medium at 25°C or 37°C for 4 h (exponential) or 16 h (stationary), and rovC transcript levels were analyzed by northern blotting. Total RNA was prepared, separated on 0.7% MOPS agarose gels, transferred onto a nylon-membrane and probed with a digoxigenin (DIG)-labeled PCR fragment encoding the rovC gene. 16S and 23S rRNAs were used as loading controls. The rovC mutant strain YP148 served as negative control; exp = exponential, stat = stationary growth. (C) Temperature-dependent expression of a translational T6SS4'-'lacZ (pSSE64) fusion in Y. pseudotuberculosis YPIII wild type was monitored and analyzed as described above. The data represent the mean ± standard deviation of three independent experiments, carried out in triplicates. Data were analyzed by Student’s t-test; *** P<0.001. (D) Expression of a translational rovC’-‘lacZ reporter fusion (pSSE32) was monitored in the Y. pseudotuberculosis wild type strain (YPIII) and the ΔrovC mutant strain (YP154). Both strains were transformed with the empty vector pACYC184 (pV) or complemented with the rovC+ overexpression plasmid pSSE11 (pProvC::rovC+). β-galactosidase activity (μmol/mg.min) was measured in strains grown over night for 16 h at 25°C in LB medium. The data represent the mean ± standard deviation of three independent experiments, carried out in triplicates. Data were analyzed by Student’s t-test. (PDF) [file ppat.1008552.s010.pdf]

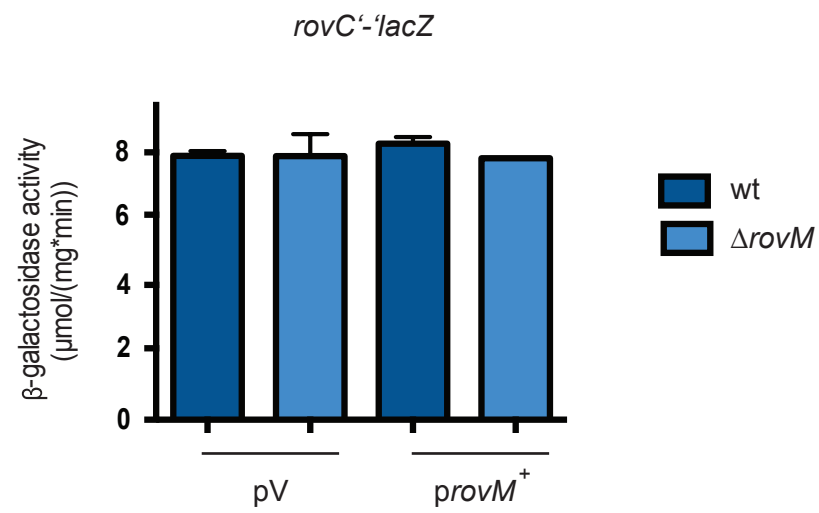

**Figure S6:** Knittel *et al.* 2020

Supplement: S6 Fig — Expression of a translational rovC’-’lacZ fusion encoded by pSSE32 was monitored in Y. pseudotuberculosis YPIII (wildtype) and the ΔrovM mutant strain (YP72) transformed with the vector pIV2 (pV) or the rovM+ plasmid (pAKH64). β-galactosidase activity (μmol/mg.min) was measured after strains were grown in LB medium at 25°C. Data are means and standard deviations of three independent experiments, each performed at least in triplicates. Data were analyzed by Student's t test. (PDF) [file ppat.1008552.s011.pdf]
