# Supplementary material for: RovC - a novel type of hexameric transcriptional activator promoting type VI secretion gene expression
Source: PLoS Pathog. 2020 Sep 23;16(9):e1008552. doi: 10.1371/journal.ppat.1008552 (PMC7535981; doi:10.1371/journal.ppat.1008552)
Supplement: S4 Fig — (A) Alignment of Y. pseudotuberculosis (Y. pstb) RovC related proteins (red: 100% conserved amino acids; orange: 80%; yellow: 60%) (WP_002224092—hypothetical protein from Y. pestis biovar mediaevalis (Y. pestis); WP_049614373—hypothetical protein from Y. pekkanenii (Y. pekka); WP_156293748—hypothetical protein from Serratia oryzae (S. oryzae); WP_161740800—hypothetical protein from Serratia fonticola (S. fonti); WP_115459941.1—hypothetical protein from Enterobacillus tribolii (E. triboli); WP_049848866—hypothetical protein from Trabulsiella odontotermitis (T. odonto); WP_038162275.1—hypothetical protein from Trabulsiella guamensis (T. guamensis); WP_162080698—hypothetical protein from Enterobacterium bacterium (E. bacter), and WP_130835776—hypothetical protein from Erwinia mediterraneensis (E. medit). Alignment generated with GeneDoc [74].]. Percentage values on the right correspond to the sequence identity to RovC of Y. pseudotuberculosis. (B) Conserved regions on the surface of RovC shown in two orientations. Color scheme as in (A). The dark blue region corresponds to the elongated stretch with ill-defined density in the structure, to which a conservation could not be mapped to. (PDF) [file ppat.1008552.s009.pdf]

A

```
Y. pstb      : -------mrkklyndfaweclrrnpqyisdwelfmkntltngggipddseliqseldlnaekkwgvmkyid : 64
Y. pestis    : -------mrkklyndfaweclrrnpqyisdwelfmkntltngggipddseliqseldlnaekkwgvmkyid : 64  99%
Y. pekka     : -------mekkiyndfaweclrrntqyirdwelfmgsnhrgtifiddkseliqseldlnaeekwglikyis : 64  79%
S. oryzae    : -------mkklvytdfaweclrrnpqyisdweiameegcyvs-gisddtartqsildleaekkwglmkyve : 63  55%
S. fonti     : -------mkktiytdfaweclrrnplyirdweivsnstpknrgkiydgqvnlqnpldlvaekkwgimkyve : 64  53%
E. tribolii  : -------mnsetytdfaweclrrnklyikdwysfknqet---------sqeyqndidliakkkwgllkfvd : 55  51%
T. odonto    : -------mdseaytdfaweylrrnkqyiadwelsnnsks---------rpgiqtildrnaekkwgllkyad : 55  50%
T. guamensis : -------mdseaytdfaweylrrnkhyiadwelsknsks---------rpgiqttldrnaekkwgllkyad : 55  49%
E. bacter    : -------mkkeiytdfaweclrrnknyinawkineskit---------enerqqhidldaeirwgllkyvn : 55  44%
E. medit     : mddrgqymenytytdiaweflrrnkdyirdwknlgrvks-geehvkrflevrqnendllaekkwgilkfid : 70  44%

Y. pstb      : pynsdptnvfwslklsnrsvrvklsntgnvkggytwgdmsnlpgvkhqrllmhdntlcvkifsqngyfqlf : 135
Y. pestis    : pynsdptnvfwslklsnrsvrvklsntgnvkggytwgdmsnlpgvkhqrllmhdntlcvkifsqngyfqlf : 135  99%
Y. pekka     : pydsdptnvfwsqelskrsvrvtlsntgkinggytwgdlsnrtgvkhqilqmhdkslcvkifnqnayfqlf : 135  79%
S. oryzae    : pgnpdpidvfwsprlskrsirlalsty----gdftwgsitgkpgvhqrtlrlmddtccikifnnnnyfqff : 130  55%
S. fonti     : pgnsdpidvfwsprlskrsirlavsts----gdftwghisdkpgvhchklrlldstmcikifnnngyfqff : 131  53%
E. tribolii  : pnhatpkgvfwspelsrrstriilsnn----gyltwgnientpnlekkklflldgslcvkvfnksnyfqff : 122  51%
T. odonto    : pseknpknvfwspelssksarvmlsky----gdciwgslykksgmnlekltlqdkttcikifnqndyfqff : 122  50%
T. guamensis : pseknpknvfwspelssksarvilsky----gdciwsslyknsgmklekltlqdkttcikifnqndyfqff : 122  49%
E. bacter    : pehsrpekvfwspklskkslpiilgkn----gdfawknivknsevrhekiflldgslcikifnhndyfqff : 122  44%
E. medit     : pdnsnpsnvfwsprlskkstriifsqs----gscswenifentrvqhkklilhdnslcikifnnheyfqff : 137  44%

Y. pstb      : iesadalkddsnlyiyiplnlesdvfaknielllqsivnhkieveckeeqylgllktiddrkqgfshrdias : 206
Y. pestis    : iesadalkddsnlyiyiplnlesdvfaknielllqsivnhkieveckeeqylgllktiddrkqgfshrdias : 206  99%
Y. pekka     : iestttlmadsnlyiyiplhlesysfsknieillqsivnhkieveakeeqylgllktiddrkqgfshrdias : 206  79%
S. oryzae    : adggitlnensnlflyiplslktntinrnidiikniinnqcetdekekqclellktiddrnqgfthkdias : 201  55%
S. fonti     : tdtstplsesnslflyiplnlqtevinknidiikgilsnrseidgkekhhmqllrtidrrkqgfthkgias : 202  53%
E. tribolii  : iddasalndrsniflyvplclnnnisnknidiikniinnkiegddkeeqyltllktvddlnkgfshraias : 193  51%
T. odonto    : iedidalnsssriflcfplnldnkiksknidfikgiinneisdlnkep-ppnlletiddlnkgfshrtias : 192  50%
T. guamensis : iedidalnsrsriflcfplnldnkiksknidfikgiinnqisdlnkep-ppnlletiddlnkgfshrtias : 192  49%
E. bacter    : isdaseltdeskiflymplslnnstrnrnidiinsifndkvetanregqqqdlldtidginegfshraias : 193  44%
E. medit     : idapvqinkrsqfyiyvpltlnndccnkcaavingilnnkskssakesrhhkllatidsmnkglshreias : 208  44%

Y. pstb      : eifgkelvknewsadswvrakiryrikkanalinygylnfl : 247
Y. pestis    : eifgkelvknewsadswvrakiryrikkanalinygyln-- : 245  99%
Y. pekka     : kifgkelvnnewsadswvrakiryrikkanelinhgylnfl : 247  79%
S. oryzae    : eifgeervrsewssdswlraniryrlkkandlitngylnyl : 242  55%
S. fonti     : elfgeeivknewssdswlraniryrlkkanklisdgyldyi : 243  53%
E. tribolii  : eifgeqlverewssdswlrsniryrikkatnlintgylnyi : 234  51%
T. odonto    : kifgkelvetewssdswlraniryrikkaitlvntgylnyi : 233  50%
T. guamensis : kifgkelvetewssdswlraniryrikkaitlvntgylnyi : 233  49%
E. bacter    : rifgeqlvesewssdswlraniryrikkainlidtgylnyl : 234  44%
E. medit     : hlfgekavekewssdswlraklryrikkanelinfgyydyl : 249  44%
```

B

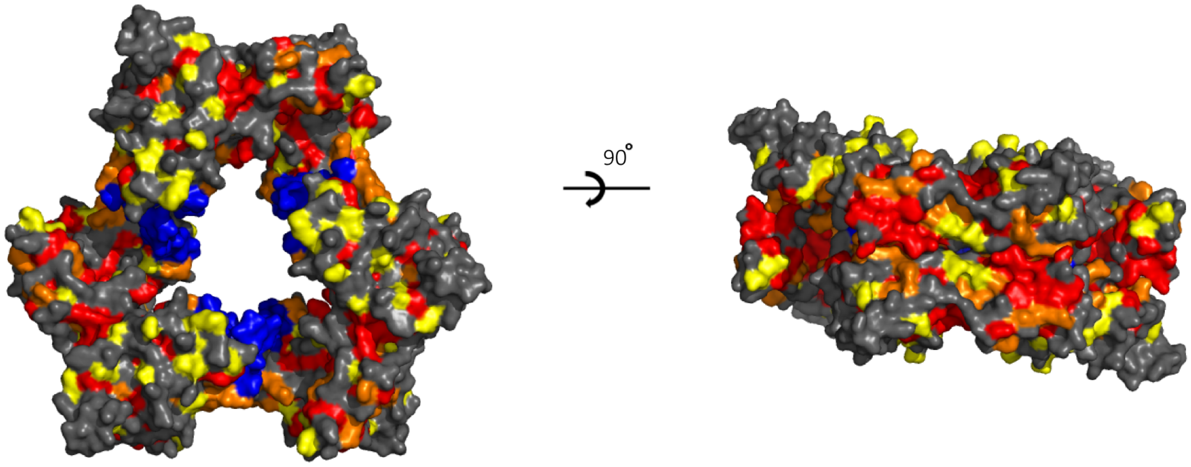

Figure S4: Knittel *et al.* 2020
